# Longitudinal normative OCT retinal thickness data for wild-type mice, and characterization of changes in the 3×Tg-AD mice model of Alzheimer's disease

**DOI:** 10.18632/aging.202916

**Published:** 2021-04-02

**Authors:** Hugo Ferreira, João Martins, Paula I. Moreira, António Francisco Ambrósio, Miguel Castelo-Branco, Pedro Serranho, Rui Bernardes

**Affiliations:** 1 University of Coimbra, Coimbra Institute for Biomedical Imaging and Translational Research (CIBIT), Institute for Nuclear Sciences Applied to Health (ICNAS), Coimbra, Portugal; 2 University of Coimbra, Coimbra Institute for Clinical and Biomedical Research (iCBR), Faculty of Medicine (FMUC), Coimbra, Portugal; 3 University of Coimbra, Center for Innovative Biomedicine and Biotechnology (CIBB), Coimbra, Portugal; 4 University of Coimbra, Clinical Academic Center of Coimbra (CACC), Faculty of Medicine (FMUC), Coimbra, Portugal; 5 University of Coimbra, Laboratory of Physiology, Faculty of Medicine (FMUC), Coimbra, Portugal; 6 University of Coimbra, Center for Neuroscience and Cell Biology (CNC), Coimbra, Portugal; 7 Universidade Aberta, Department of Sciences and Technology, Lisboa, Portugal

**Keywords:** optical coherence tomography, normative data, Alzheimer's disease, mouse model, retinal thickness

## Abstract

Mice are widely used as models for many diseases, including eye and neurodegenerative diseases. However, there is a lack of normative data for retinal thickness over time, especially at young ages. In this work, we present a normative thickness database from one to four-months-old, for nine layers/layer-aggregates, including the total retinal thickness, obtained from the segmentation of spectral-domain optical coherence tomography (SD-OCT) data from the C57BL6/129S mouse strain. Based on fifty-seven mice, this normative database provides an opportunity to study the ageing of control mice and characterise disease models' ageing, such as the triple transgenic mouse model of Alzheimer's disease (3×Tg-AD) used in this work. We report thickness measurements, the differences in thickness per layer, demonstrate a nasal-temporal asymmetry, and the variation of thickness as a function to the distance to the optic disc centre. Significant differences were found between the transgenic group's thickness and the normative database for the entire period covered in this study. Even though it is well accepted that retinal nerve fibre layer (RNFL) thinning is a hallmark of neurodegeneration, our results show a thicker RNFL-GCL (RNFL-Ganglion cell layer) aggregate for the 3×Tg-AD mice until four-months-old.

## INTRODUCTION

Optical coherence tomography (OCT) has revolutionized the imaging of the eye [1]. It allows the imaging of human and animal eyes, *in vivo* and *in situ*, with high-resolution, allowing the study of the eye both in its healthy and diseased conditions [2, 3], being the retina the most explored structure, to date, using this technique.

Mouse models of disease are fundamental in many scientific fields, including eye-related conditions, and allow broadening our understanding of the pathophysiology of these [4–6]. Historically, the study of mouse retinas has been mostly done *ex vivo*, limiting the number of study-cases and denying the possibility of following the same set of individuals over time. OCT provides an *in vivo* imaging solution allowing to overcome these limitations and expanding further the usefulness of mice models of disease.

Remarkably, a literature search shows a lack of retinal thickness normative data for mice. For the commonly used mouse strain (C57BL/6), a paper can be found reporting the average thickness for several layers, and the total retinal thickness (TRT), for 30 mice aged from three to five-months-old [2].

The main goals of this work are, to report normative thickness data for both the C57BL6/129S (Wild-Type; WT) and the triple transgenic (3×Tg-AD) mice, harbouring three human genes: Swedish amyloid precursor protein (APPswe), presenilin 1 (PSEN1), and microtubule-associated protein tau (MAPT), which are associated with familial Alzheimer's disease. Results herein are supported by the unusually high number of mice, the matching of mice's ages, and the monthly follow-up from the age of one to four-months-old. Here, we report the average TRT and the average retinal thickness for individual retinal layers. We also demonstrate a nasal-temporal asymmetry concerning the retinal thickness and report the variation of thickness as a function to the distance to the optic disc centre, all based on detailed thickness maps composed of 512x512 values each.

## MATERIALS AND METHODS

### Ethics statement

This study was approved by the Animal Welfare Committee of the Coimbra Institute for Clinical and Biomedical Research (iCBR), Faculty of Medicine, University of Coimbra. All procedures involving mice were conducted as per the Association for Research in Vision and Ophthalmology statement for animal use, and in agreement with the European Community Directive Guidelines for the care and use of nonhuman animals for scientific purposes (2010/63/EU), transposed into the Portuguese law in 2013 (DL113/2013).

### Mouse characterization

Fifty-seven male mice from each strain, C57BL6/129S and 3×Tg-AD, were used, and both eyes imaged at the ages of one, two, three and four-months-old. Mice from both groups were enrolled in the study within nine months as they became available. All mice were housed and maintained at the vivarium of the Coimbra Institute for Clinical and Biomedical Research (iCBR), Faculty of Medicine, University of Coimbra, and were on a 12-h light/dark cycle with free access to both food and water.

### Experimental setup

Concerning OCT imaging preparation, mice were anaesthetized using a mixture of 80 mg/kg of ketamine (Nimatek; Dechra) and 5 mg/kg of xylazine (Sedaxylan; Dechra). The pupils were dilated using a solution of 0.5% tropicamide (Tropicil; Edol) and 2.5% phenylephrine (Davinefrina; Dávi). Additionally, oxibuprocaine (Anestocil; Edol), a local anaesthetic, was used. Eyes were regularly lubricated using eye drops (1% carmellose (Celluvisc; Allergan)).

**Figure 1:**
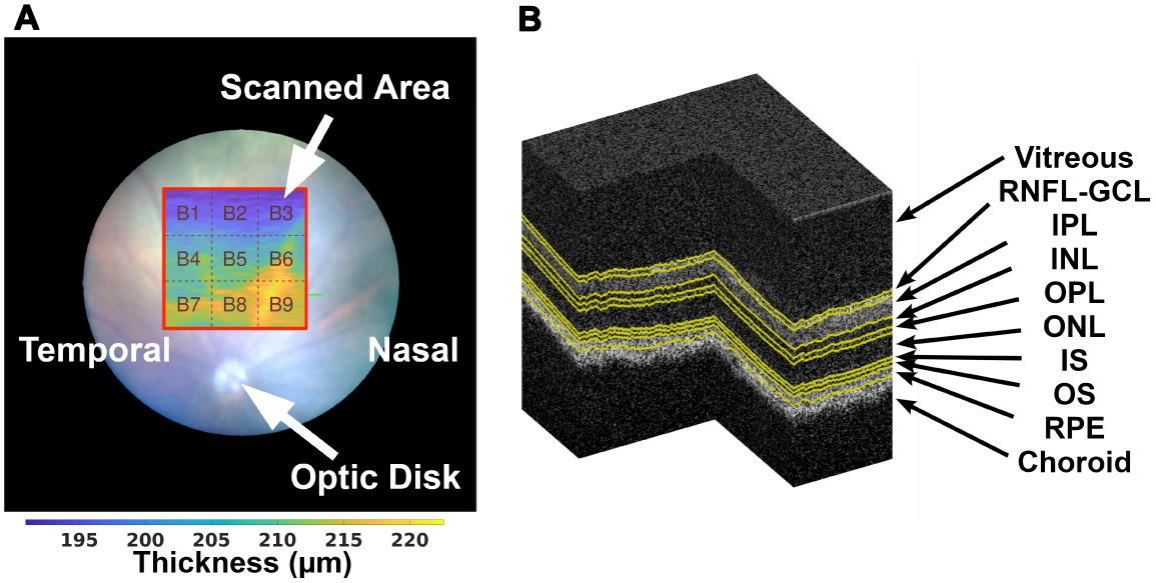
On the left (**A**), the illustration of the scanned area (with red border) with relation to the position of the optic disc, and division of the thickness maps into 3×3 blocks as addressed in section 2.5, over a TRT thickness map from a WT mouse at the age of one-month-old. A segmented OCT volume is shown on the right (**B**), where the various interfaces are presented in yellow.

All retinas were imaged by a Micron IV OCT System (Phoenix Technology Group, Pleasanton, CA, USA). It creates a volume per acquisition composed of 512 B-scans, each with 512 A-scans of 1024 pixels in length; B-scans are saved as a non-compressed TIFF file image. The system presents an imaging depth of 1.4 mm and an axial resolution of 3 μm, as determined by the bandwidth and central wavelength, respectively 160 and 830 nm, of the superluminescent diode used. All scans were taken by the same operator (JM) in the same retinal region using the optic disc as a landmark; centered horizontally with the optic disc, and vertically above it (Figure 1A).

### Segmentation

In this work, the OCT data is used for the segmentation of eight distinct layers, allowing the determination of the following: retinal nerve fibre layer and ganglion cell layer complex (RNFL-GCL), inner plexiform layer (IPL), inner nuclear layer (INL), outer plexiform layer (OPL), outer nuclear layer (ONL), the inner segment of the photoreceptors (IS), the outer segments of the photoreceptors (OS), and the retinal pigmented epithelium (RPE).

The segmentation is achieved using a fully convolutional neural network (FCNN), following a U-type architecture [7], which is composed of two main parts, an encoding path and a decoding path. Each level reduces the feature maps' size in the encoding path, effectively computing increasingly global features. The decoding path takes these global features and expands them to classify individual pixels in one of the layers considered in this study. Furthermore, these neural networks can have short-circuit connections [7]. These pathways connect encoding and decoding levels allowing the use of local features, obtained along the encoding path, to provide finer grain information and produce more accurate pixel classification predictions. The neural network model used, with the characteristics detailed above, is disclosed in [8].

In brief, while the network was designed to classify pixels into one of the retina's eight layers, the main objective is to ensure the proper discrimination at these layers' interfaces. The ground-truth interface samples were obtained based on segmented volumes (Figure 1B).

Because of the limited training set and to eliminate the possibility of the network to learn from the usual location of retinal layers within B-scans, the training set was augmented in two ways: by mirroring each B-scan horizontally, and by modulating the location of the retina across the B-scan image. The latter was achieved by circularly-shifting each A-scan across the B-scan by an amount given by a sinusoid which parameters, amplitude, frequency and phase, were randomised per B-scan. The segmentation provided by the neural network was then validated using manual segmentation examples provided by two graders (HF and Raquel Boia).

### Thickness

The segmentation of the entire volume (Figure 1B), is achieved by segmentation of each of the 512 B-scans. All thickness maps, per retinal layer and the TRT, are computed as the distance between respective boundaries and defined by 512×512 thickness values.

Due to the nasal-temporal asymmetry found between left and right eyes, all left-eyes were mirrored to match the right ones to ease comparisons. As such, left-hand-side/right-hand-side to thickness maps corresponds to the temporal/nasal region, respectively.

Besides the possibility of disclosing the average retinal thickness for the entire imaged region of the eye, thickness maps of 3×3 values were computed as the average thickness of 170×170 thickness values of the original 512×512 thickness maps (Figure 1A) after cropping it to 510×510 pixels so that all blocks are of equal size.

Average thickness is presented both for the entire imaged area and for 3×3 blocks. The former is the standard measure disclosed in the literature, while the latter provides some data regarding thickness variation over the scanned area.

### Statistical analysis

Individual thickness values, each of the 512×512 values of each thickness map, were assessed for their segmentation quality based on multiple criteria including image quality, segmentation (boundary) consistency, and computed thickness values distribution. Those not fulfilling one or more of the established criteria were individually excluded from all analysis.

For establishing the reference thickness maps, only blocks with 90%, and above, of the 170×170 thickness values fulfilling the above criteria were considered. Using these criteria, the percentage of blocks removed was 9.8%, 4.8%, 10.7%, 1.2%, 0.7%, 1.0%, 0.7%, 0.7% and 0.9%, respectively for blocks one to nine, as in Figure 1A.

The Kolmogorov-Smirnov normality test was applied to each group, finding that the large majority of distributions do not reject the null hypothesis that the distributions are normal. Hence, the t-test was used to determine significant statistical differences between WT and 3×Tg-AD groups. Significance levels of 5%, 1%, and 0.1% were used in this work.

For the analysis of the thickness data over time, at the ages of one, two, three, and four months, the repeated measures ANOVA (RANOVA) was performed. The same analysis was performed to study differences between each block in the 3×3 thickness map. The multiple comparisons corrections performed in this work used the Tukey test.

All data processing and statistical treatment were performed using Matlab R2020a (The MathWorks Inc., Natick, MA, USA).

## RESULTS

The fifty-seven mice were available at the start of this study. However, due to subpar acquisition quality, segmentation errors, and mice deaths, the actual number of volumes per eye and time point vary. Table 1 shows the actual number of acquisitions used for the results presented next.

**Table 1:** Number of volumes by group, eye and age.

**Group**	**Eye**	**One month**	**Two months**	**Three months**	**Four months**
WT	OS	54	52	49	53
	OD	50	50	36	50
3×Tg-AD	OS	46	41	43	40
	OD	48	42	45	44

### Weight

As shown in Table 2, weight consistently increases over the reported period for both WT and 3×Tg-AD mice. Furthermore, the highest increase in weight occurs between one and two-months-old, as the mice reach their maturity at the ages of six to eight weeks, after which growth slows down.

**Table 2:** Weight distribution for each of the studied groups over the four time-points (mean (std)) (in grams).

**Group**	**One month**	**Two months**	**Three months**	**Four months**
WT	14.84 (2.68)	22.65 (1.69)	25.29 (1.92)	27.02 (2.07)
3×Tg-AD	14.94 (2.66)	22.33 (2.31)	25.57 (2.27)	27.41 (2.06)
p-value	0.86	0.39	0.47	0.36

Comparing weight distributions for both groups did not find statistically significant differences between groups at any of the reported ages.

### Thickness maps

**Figure 2:**
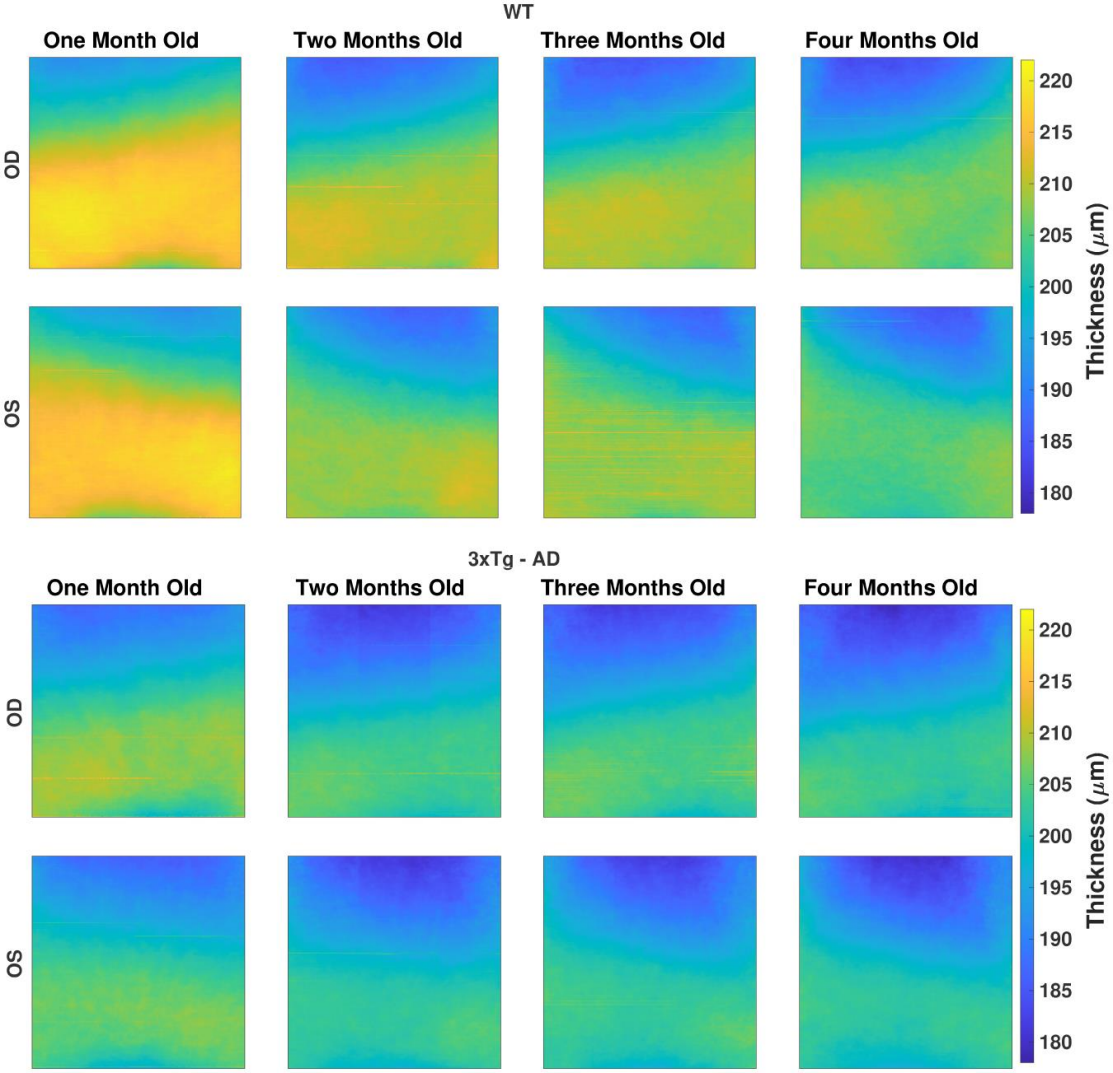
**The average total retina thickness map (in μm) for both WT (top) and 3×Tg-AD group (bottom), separated by eye (rows), right (OD) and left eyes (OS), and by age (columns).** The color range is consistent for all maps.

Detailed average thickness maps of 510×510 values were computed for the right and left-eyes per each retinal layer and the TRT. The latter is shown in pseudocolor in Figure 2, for WT and 3×Tg-AD groups, at each time point.

Besides the apparent nasal-temporal asymmetry, thickness values are within the same range for both eyes of the same mice group at the same time point. Indeed, a thicker nasal side was found with statistically significant differences to the temporal side for all layers/layer-aggregates, except for the IPL, in three or more time points for the right-eye of WT mice.

Over time, the thickness difference between the ages of one and two-months-old stands out as notoriously greater than the thickness difference between the remaining consecutive ages. Nevertheless, while the TRT showed a consistent decrease over time, individual layer thickness behavior differs, as shown ahead in this section.

### Mean thickness

Tables 3, 4 show the average (and standard deviation) thickness values, per retinal layer, for WT and 3×Tg-AD mice, combining the data from both eyes, for the entire imaged area (Figure 1A). For detailed data, please refer to Supplementary Tables 1–4.

**Table 3:** Thickness values (m(sd)) (in μm) for both eyes of wild-type mice, at the ages of one, two, three and four-months-old.

	**One month**	**Two months**	**Three months**	**Four months**
	**(N=104)**	**(N=102)**	**(N=85)**	**(N=103)**
RNFL-GCL	12.90 (0.72)	13.10 (0.89)	13.44 (0.93)	13.48 (0.72)
IPL	51.55 (2.04)	48.04 (2.31)	47.52 (3.19)	47.23 (1.92)
INL	25.57 (1.02)	21.93 (0.86)	21.45 (0.89)	20.62 (0.61)
OPL	15.22 (0.30)	15.13 (0.31)	15.12 (0.21)	15.11 (0.23)
ONL	62.13 (1.17)	60.64 (1.38)	60.64 (1.19)	60.15 (0.98)
IS	10.79 (0.53)	11.07 (0.57)	11.04 (0.42)	11.21 (0.42)
OS	11.61 (0.40)	11.36 (0.47)	11.19 (0.33)	11.14 (0.39)
RPE	20.93 (1.45)	22.89 (1.04)	23.24 (1.23)	23.47 (0.90)
TRT	210.06 (3.09)	202.90 (3.29)	203.34 (3.59)	200.69 (2.40)

The significant reduction in the number of acquisitions at three-months-old was due to sub-par image quality.

**Table 4:** Thickness values (m(sd)) (in μm) for both eyes of 3×Tg-AD mice, at the ages of one, two, three and four-months-old.

	**One month**	**Two months**	**Three months**	**Four months**
	**(N=94)**	**(N=83)**	**(N=88)**	**(N=84)**
RNFL-GCL	13.15 (0.79)	13.69 (0.87)	13.95 (0.85)	14.03 (0.72)
IPL	47.92 (2.45)	45.59 (2.39)	45.79 (2.30)	45.86 (2.72)
INL	22.41 (0.99)	19.63 (0.85)	19.50 (0.62)	18.96 (0.73)
OPL	14.90 (0.31)	14.81 (0.26)	14.92 (0.23)	14.89 (0.27)
ONL	62.09 (1.58)	60.57 (1.80)	60.59 (1.64)	59.86 (1.84)
IS	10.18 (0.36)	10.61 (0.38)	10.80 (0.40)	10.97 (0.36)
OS	11.29 (0.38)	11.34 (0.50)	11.34 (0.40)	11.43 (0.41)
RPE	19.42 (0.82)	21.46 (1.06)	21.90 (1.01)	22.29 (1.02)
TRT	200.54 (3.83)	196.77 (3.70)	197.68 (3.18)	196.16 (3.14)

Comparing right to left eyes within groups found no statistically significant differences. On the other hand, as illustrated in Tables 3, 4, the comparison between groups shows consistent retinal layer differences. Overall, the 3×Tg-AD group's eyes present a decreased layer thickness except for the RNFL-GCL complex, where the 3×Tg-AD group present an increased thickness, and the ONL, where thickness values match one another.

Statistically significant differences between groups can be found in Table 5 for both eyes. While the highest statistical differences were found for the IPL, INL, OPL, IS, RPE and the TRT, no differences were found for the ONL at any of the significance levels considered in this work. The RNFL-GCL complex showed statistically significant differences starting at the age of two-months-old on both eyes. On the other hand, the OS layer presents statistically significant differences at the age of one and four-months-old for both eyes.

**Table 5:** Significant difference levels between the WT and 3×Tg-AD groups, for right (OD) and left (OS) eyes, by retinal layer and time point.

		**One month**	**Two months**	**Three months**	**Four months**
Right Eyes (OD)	RNFL-GCL	-	■	■	●
	IPL	*	*	●	■
	INL	*	*	*	*
	OPL	*	*	*	*
	ONL	-	-	-	-
	IS	*	*	●	■
	OS	*	-	-	*
	RPE	*	*	*	*
	TRT	*	*	*	*
Left Eyes (OS)	RNFL-GCL	-	*	●	*
	IPL	*	*	*	■
	INL	*	*	*	*
	OPL	*	*	*	*
	ONL	-	-	-	-
	IS	*	*	■	■
	OS	*	-	-	*
	RPE	*	*	*	*
	TRT	*	*	*	*

The results follow the common definition with significance levels at p-value *<* 0.05 (●), p-value < 0.01 (■) and p-value *<* 0.001 (*).

Presented data also shows the consistent decrease in thickness for the INL and IPL, respectively of 20% and 10% across the timespan covered, which was partially counterbalanced by the consistent increase of the RPE layer thickness of about 15% for the same period.

**Figure 3:**
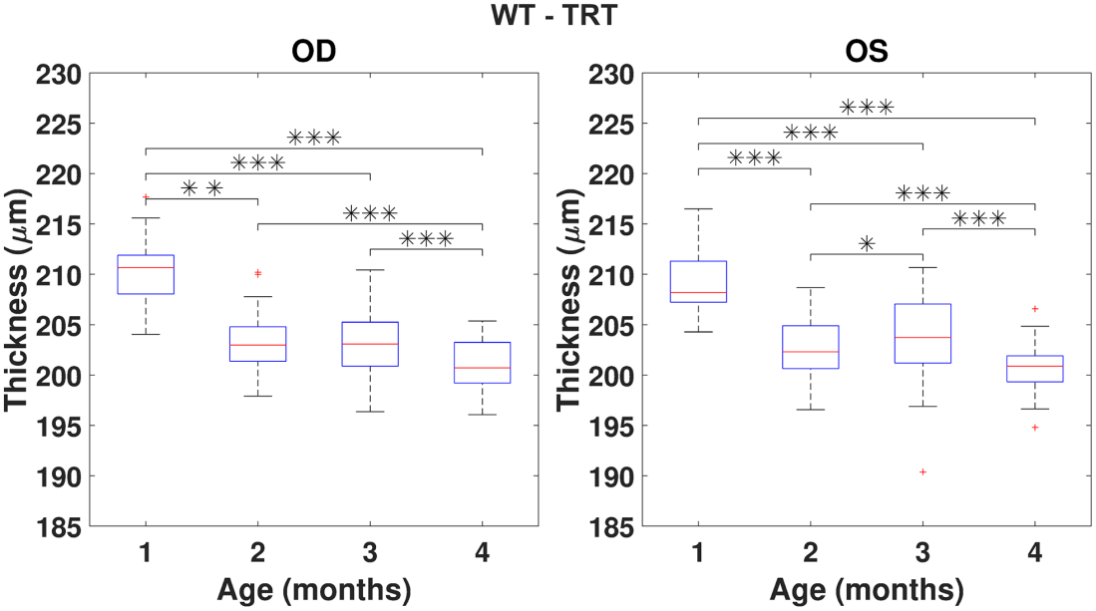
**Boxplot of the total retinal thickness (TRT) (in μm) for each time point, for right (OD) and left (OS) eyes of WT mice (respectively, left and right graphs).** One, two and three asterisks represent, respectively, statistically significant differences at the level of 5%, 1% and 0.1%, based on pairwise comparisons. No statistically significant differences were found when comparing right to left eyes at any time point.

**Figure 4:**
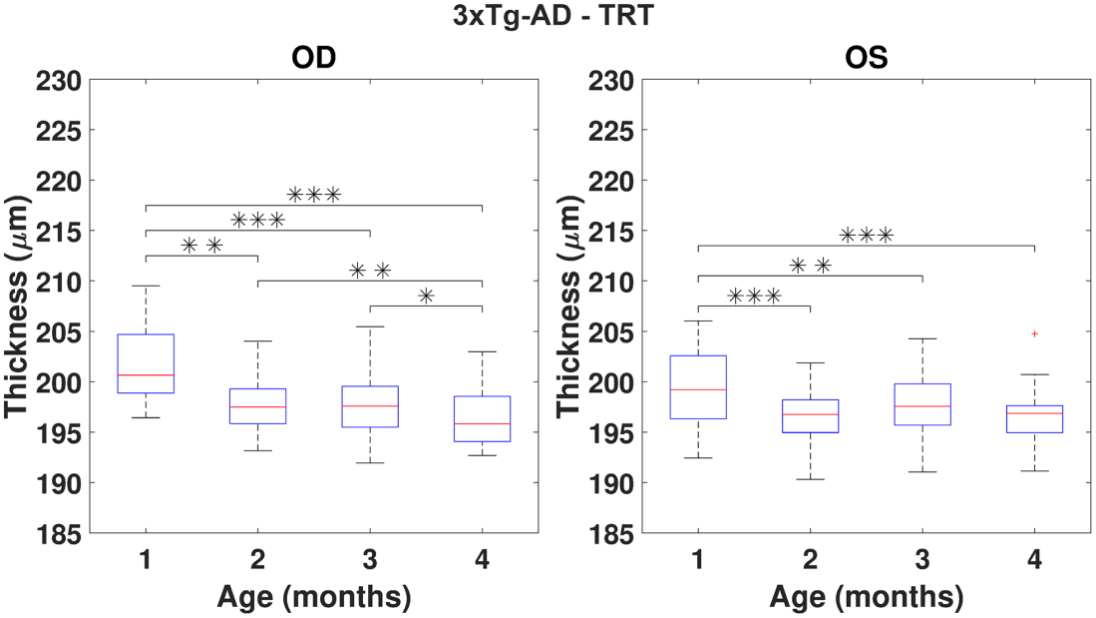
**Boxplot of the total retinal thickness (TRT) (in μm) for each time point, for right (OD) and left (OS) eyes of 3×Tg-AD mice (respectively, left and right graphs).** One, two and three asterisks represent, respectively, statistically significant differences at the level of 5%, 1% and 0.1%, based on pairwise comparisons. No statistically significant differences were found when comparing right to left eyes at any time point.

Using the repeated measures ANOVA (RANOVA), the thickness distributions were compared across the four-time-points for each group/eye combinations. Only eyes successfully imaged and processed at all four-time-points (WT (N=37) and 3×Tg-AD (N=35)) were considered for this particular analysis. Achieved results show thickness to be dependent on age for the period covered in this study. Pairwise comparisons are illustrated in Figures 3, 4 for the TRT, respectively for the WT and 3×Tg-AD groups. Statistically significant differences were found between the first and remaining time points for all eyes. Also, except for the left-eye of the 3×Tg-AD group, all remaining eye present statistically significant differences between the four and all remaining time points. For detailed data, please refer to Supplementary Figures 1–16.

### Normative thickness map

In addition to the above mean thickness values, we also present the average thickness (and standard deviation) for each layer, for each of the blocks (as in Figure 1A), for OD and OS eyes of WT mice, Supplementary Tables 5–8. Likewise, based on the asymmetry found between right and left eyes, thickness values obtained by combining data from both eyes are also shown in Supplementary Tables 5–8. It is noteworthy that the TRT thickness presented in these Tables does not need to exactly match the sum of the thickness of the remaining layers, as the block selection process detailed in section 2.6 is done independently for each layer.

Because most thickness values present a normal distribution for all the nine blocks, all retinal layers at hand in this study and the four-time-points, average and standard deviation values for each block are enough to establish the normative thickness distribution. Furthermore, it allows computing the probability (p-value) of a particular thickness value to be out of the normal range. Section 3.5 will explore further this subject.

The normative data presented in Supplementary Tables 5–8 demonstrates the distance dependence of retinal thickness to the optic disc. In general, thickness decreases as the distance to the optic disc increases. However, the OPL, in addition to the IS, and OS layers, presents a homogenous thickness across the entire imaged region.

Even though the average thickness values for the entire imaged region have shown evident thickness differences over time, averaging smaller regions allows for a better picture of the retina's ongoing changes. For instance, while the average OPL thickness did not show any difference in thickness over time, its detailed analysis shows a decrease in thickness in regions further away from the optic disc counterbalanced by the increase in thickness in regions closer to it.

### Use of normative data

In the previous section, the normative thickness map distribution was established for each of the eight retinal layers assessed in this work and the TRT. In the current section, we use those normative thickness maps to show their usefulness in evaluating individual retinas.

Following the acquisition of OCT data and the segmentation of the layers of interest, computed thickness values for the 3×3 thickness map can be matched with the respective normative data to determine either the increased or decreased thickness and the probability (p-value) of the normative distribution to generate a more extreme value than the given thickness, for each of the considered four-time-points. The p-value is determined by the established normal thickness distribution for each block based on mean and standard deviation values. It relates the considered thickness with the normative distribution, being one (p=1.00) for the mean value and decreasing as values get further away from it towards the normal distribution tails.

**Figure 5:**
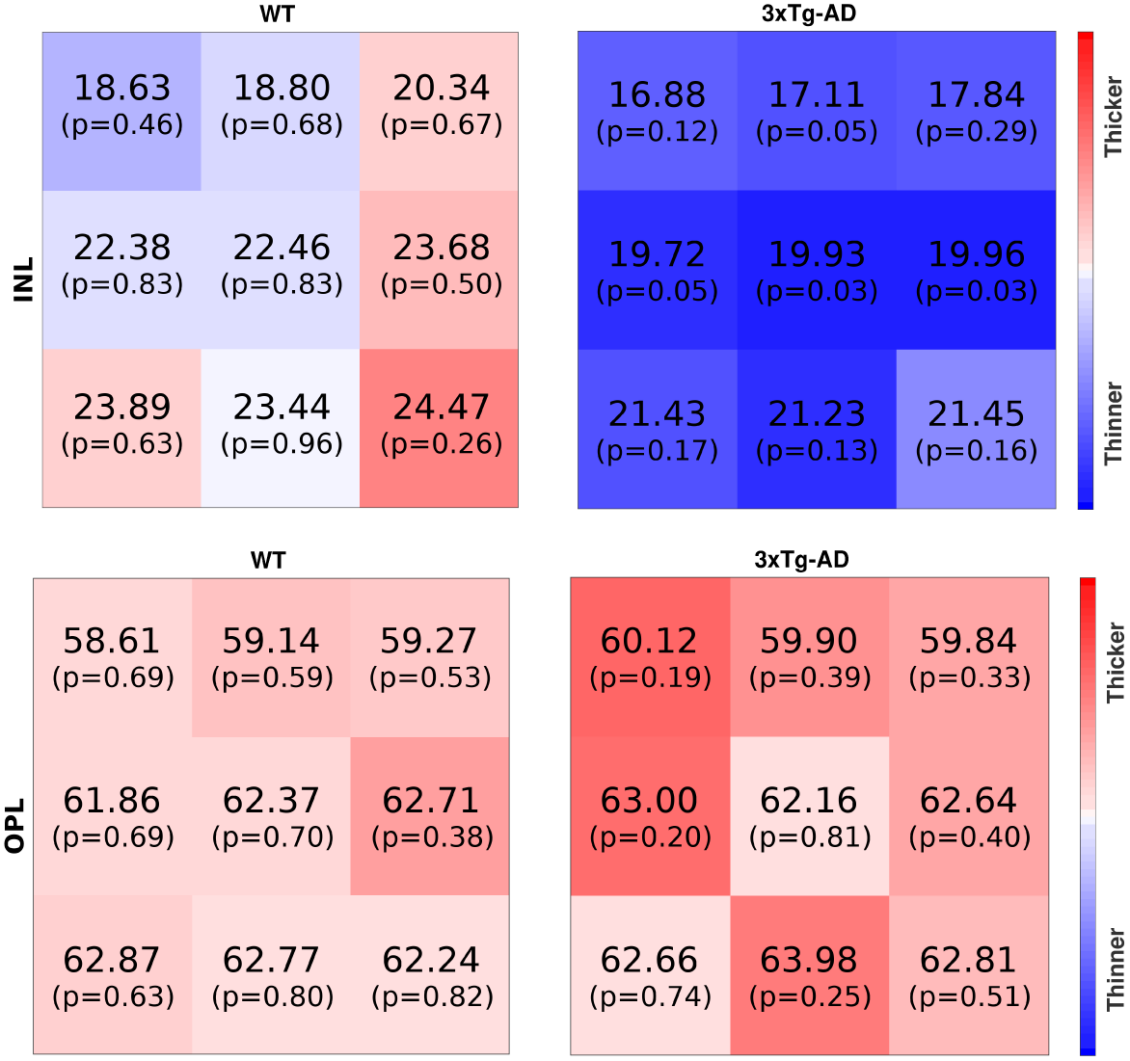
**The first row shows inner nuclear layer thickness maps for one WT (mouse E038, two-months-old, right eye) and one 3×Tg-AD (mouse E032, two-months-old, left eye), respectively the left and right maps.** The second row shows thickness maps from the outer nuclear layer for the same mouse. The thickness (μm) and p-value are shown per block. Blue/red colors denote thickness below/above the average. Color intensity increases with the increase in the probability of thickness being abnormal.

Two examples, one for WT and one for the 3×Tg-AD, at the age of two-months-old, can be seen in Figure 5, respectively for the INL and the OPL.

For the ease of interpretation, blue and red colors represent thickness values below and above the average thickness; color intensity increases with the probability of respective thickness values being extreme.

The intense blue color of the INL thickness of the 3×Tg-AD mice shown in Figure 5 indicates the overall decrease in thickness compared to the established normative data. Indeed, five out of the nine values present p-values under 0.1, meaning that the probability of randomly generating that or a more extreme thickness value for the normative distribution of the thickness is below 10%. On the other hand, for the WT thickness map, one can find both blueish and reddish colors, meaning that the thickness is around the average, respectively below and above it, with the light colors indicating these are close to the average; six of the nine values present a p-value above 0.6.

On the other hand, both maps for OPL present lower color intensities than the previous comparison, meaning that the two eyes present thickness values closer to the normal range for this layer, as conveyed by the p-values reported. This is in agreement with the above findings for this retinal layer, for which no statistically significant differences were found between groups.

## DISCUSSION

While mice have been widely used as models for many diseases, including eye and neurodegenerative diseases, and OCT is increasingly used for imaging the ocular fundus both in human and animal studies, no normative database currently exists for the thickness of the retina for several time points.

To the best of our knowledge, this is the first study to address a detailed thickness analysis based on OCT imaging of the ocular fundus of both WT and 3×Tg-AD mice in a consistent follow-up from the ages of one to four-months-old. On the one hand, this study allows for the longitudinal characterization of the healthy ageing of wild-type C57BL6/129S mice and the establishment of normative data for the retina thickness. On the other hand, it allows the characterization of pathological changes in 3×Tg-AD mice and explores differences to the control group.

In this study, we report the thickness for several layers/layer-aggregates for the ages of one, two, three, and four-months-old for both eyes of a set of 57 WT mice. Furthermore, we establish normative thickness maps for nine layers/layer-aggregates and provide average thickness values for the entire imaged area and average thickness values for nine blocks for reference.

While no statistically significant differences were found between right and left eyes, a nasal-temporal asymmetry, with statistically significant differences in thickness, was found despite all acquisitions having been performed at the same retinal location; above the optic disc and horizontally centered to it.

Interestingly, not all layers/layer-aggregates show the same behavior over time. While some show an increase in thickness, some decrease, and others remain the same over the reported period. This behavior reinforces the need to report thickness values for individual layers and not only for the total retina or substantial layer-aggregates.

Our thickness values are in line with those from Ferguson et al. [2] where the authors report thickness values for a set of 30 C57BL/6 mice three to five-months-old. Despite the differences in the imaged region, our average (standard deviation) total retinal thickness of 203.34 (3.59) microns (three-month-old mice) and 200.69 (2.40) microns (four-month-old mice), compares to the 202.74 (4.85) microns disclosed for the superior region of their study.

In a later study, the same authors report thickness for a set of 48 C57BL/6 mice three to five-months-old [9]. In this study, a further detailed set of thickness measurements allow us to show that our results are in line with their reported values considering the differences in the imaged region of the ocular fundus. Specifically, for the aggregate of the inner segment, the outer segment, and the retinal pigment epithelium (IS+OS+RPE) they disclose a thickness of 41.55 microns [9] (incorrectly mentioned as OS/IS/ELM) and 44.27 microns in [2] (adding the thickness values for the RPE and the OS/IS/ELM aggregate). In comparison, we have found 45.82 microns for the same aggregate. Also, our ONL thickness of 60.15 microns is in line with their reported value of 60.75 microns. The same verifies for the OPL for which Ferguson found a thickness of 15.80 microns and we have found a thickness of 15.11 microns.

Our results are also in agreement with those from Berger et al. [10] where spectral-domain OCT was used to image six to 12-weeks-old wild-type C57BL/6 mice.

In another study, Kim et al. [11] report data from seven WT mice, from 14 to 28 post-natal days. While reported mice's body weight matches ours, the authors reported thinner retinas. Furthermore, for the reported period, the total retinal thickness is consistently decreasing, which is in line with our findings. The decrease in thickness shown in their Figure 2B shows a much faster pace, suggesting that the retina's thinning is faster immediately after birth and slower afterwards. This finding agrees with the rapid eyeball growth up to 40 post-natal days and the slower expansion after that up to 300 post-natal days [11].

Our results are also in line with those of Chidlow et al. [12] which, for a similar study involving 50 WT and 50 double transgenic mice models of the AD, from the ages of three to twelve months, did not find differences between groups for the ONL. On the other hand, while we have found statistically significant differences for all the remaining layers in our study, these authors did not find any for the INL, IPL, or OPL layers. Unfortunately, they did not study either the RNFL, the GCL or their aggregate.

While, in general, our 3×Tg-AD mice group presents a thinner retina compared to the control group for the covered period, the RNFL-GCL aggregate presents the opposite behavior with the 3×Tg-AD group presenting a consistently thicker RNFL-GCL aggregate. For this relatively young group of animals, this finding goes on the opposite direction of a similar study from Song et al. [13] for a different age group. In their work, statistically significant thinning of the nerve fibre layer was found. In their study, Song and co-workers used 3×Tg-AD mice, but female mice were used, all beyond 15 months-old. While these findings are consistent with those found in the literature [14–16] for the human retina, the reason for the opposite behavior of our findings may lay in our much younger group of mice. Simultaneously, those from the Song's study may better mimic the human condition where the disease may progress undetected for over a decade. In this scenario, the apparent contradiction may not exist. Our results may only suggest an initial inflammation response [17] that may lead to cell death and the consequent reduction in thickness later on. Furthermore, this finding may be in line with contradictory results in humans. In the study from Sánchez et al. [18], a large study involving 930 individuals (414 cognitively healthy individuals, 192 probable amnesiac MCI and 324 probable AD), only a non-significant decrease in mean RNFL was found, which may be explained by the potential differences in stages of disease development, which our findings would support.

Finally, although Harper et al. [17] did not disclose thickness values to establish the comparison between studied groups, the trend of decreasing thickness with age was found for both the control and mouse model of the AD, which also verifies in our study. The much slower thinning than ours may be justified by the use of older mice (45 to 104 weeks).

## CONCLUSIONS

In this paper, we set a normative database for the thickness of WT C57BL6/129S mice at the ages of one to four-months-old, by monthly imaging both eyes of 57 mice and segmenting eight layers/layer-aggregates and the total retina. While the thickness of the several layers is location-dependent, specifically, dependent on the distance to the optic nerve head and dependent on age, we demonstrated that our results are in line with several studies, even though, to the best of our knowledge, no other study has mapped the thickness of mouse retina with the level of detail herein presented.

Based on the collected data, we demonstrated different behaviors for the retina's layers with ageing. While some get thicker over time, some get thinner, and some keep the same for the reported period, establishing a thickness normative database for each layer, location and time point that can be used for other comparative studies.

The same analysis was carried out on 3×Tg-AD mice model for the same period and with acquisitions performed for 57 mice at the same ages as controls. Besides the characterization of the mouse model of AD at the ages of one to four-months-old, we have demonstrated that the RNFL-GCL aggregate is thicker than that of controls. Nevertheless, the relatively younger age of mice in this study, compared to others, may explain this apparent contradiction to what is found in the literature. This finding allows us to hypothesize that we should be looking for an increase in thickness and not thinning the RNFL/RNFL-GLC aggregate at the AD's early stages.

These animals will be further monitored to assess the effect of disease progression and shed light on later changes in which we expect to find the retina's normal documented behavior compared to the controls, that is, a decrease in thickness of the RNFL-GCL aggregate.

## Supplementary Material

Supplementary Figures

Supplementary Tables 1 to 4

Supplementary Table 5

Supplementary Table 6

Supplementary Table 7

Supplementary Table 8
