# Supplementary material for: Longitudinal normative OCT retinal thickness data for wild-type mice, and characterization of changes in the 3×Tg-AD mice model of Alzheimer's disease
Source: Aging (Albany NY). 2021 Apr 2;13(7):9433–54. doi: 10.18632/aging.202916 (PMC8064224; doi:10.18632/aging.202916)
Supplement: Supplementary Figures [file aging-13-202916-s001.pdf]

SUPPLEMENTARY FIGURES

Layer thickness boxplots

The TRT thickness's boxplots are displayed in Figures 3, 4, for WT and 3×Tg-AD groups. The same boxplots, for each layer, are presented in this appendix.

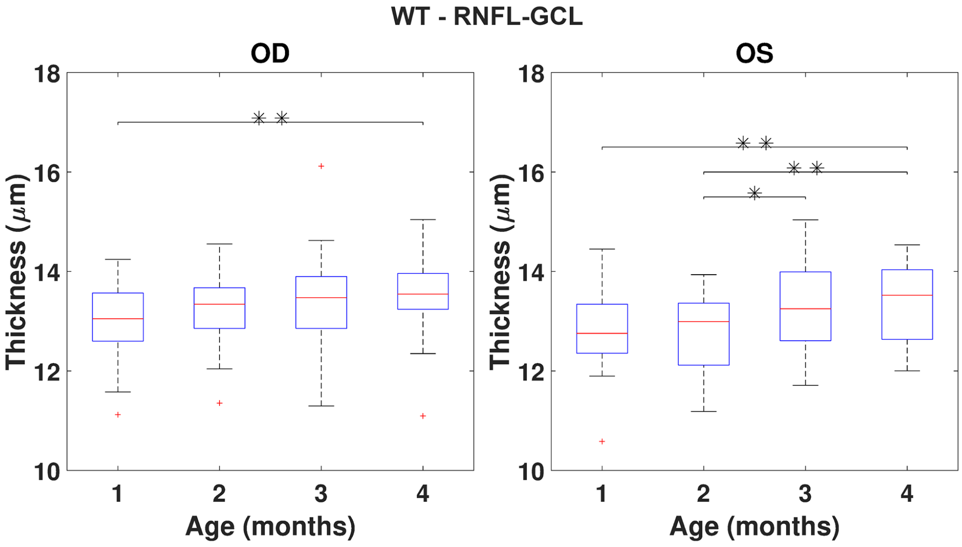

Supplementary Figure 1. Boxplot of the RNFL-GCL complex thickness (μm) for each time point, for right and left eyes of WT mice (respectively, left and right graphs). One, two and three asterisks represent, respectively, statistically significant differences at the level of 5%, 1% and 0.1%, based on pairwise comparisons.

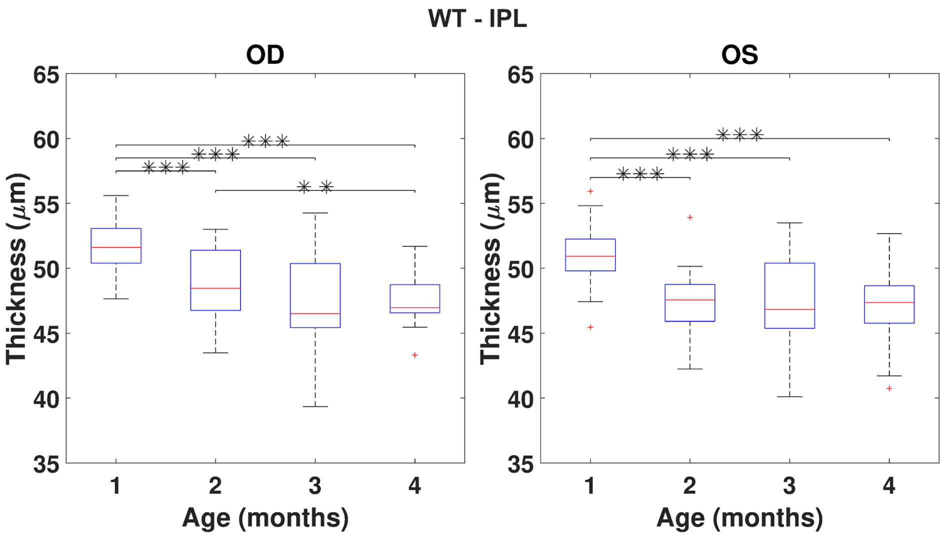

Supplementary Figure 2. Boxplot of the IPL thickness (μm) for each time point, for right and left eyes of WT mice (respectively, left and right graphs). One, two and three asterisks represent, respectively, statistically significant differences at the level of 5%, 1% and 0.1%, based on pairwise comparisons.

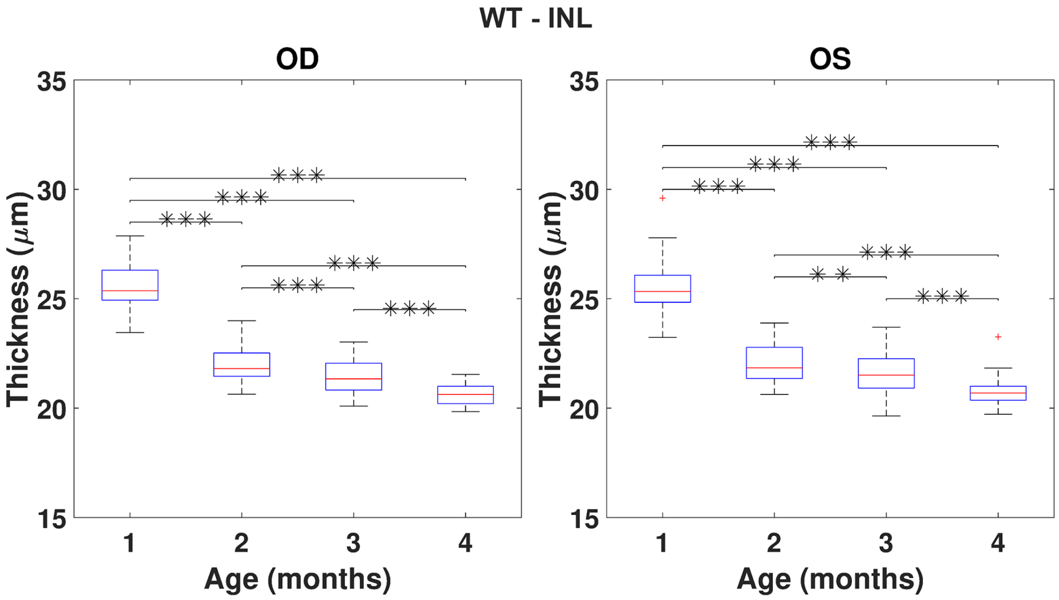

Supplementary Figure 3. Boxplot of the INL thickness (μm) for each time point, for right and left eyes of WT mice (respectively, left and right graphs). One, two and three asterisks represent, respectively, statistically significant differences at the level of 5%, 1% and 0.1%, based on pairwise comparisons.

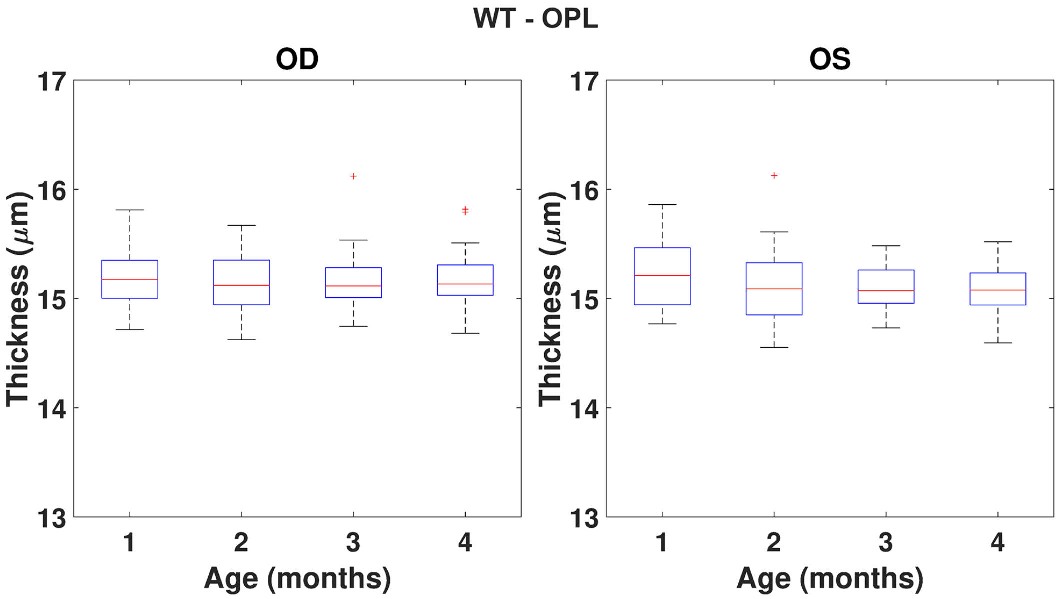

Supplementary Figure 4. Boxplot of the OPL thickness (μm) for each time point, for right and left eyes of WT mice (respectively, left and right graphs). One, two and three asterisks represent, respectively, statistically significant differences at the level of 5%, 1% and 0.1%, based on pairwise comparisons.

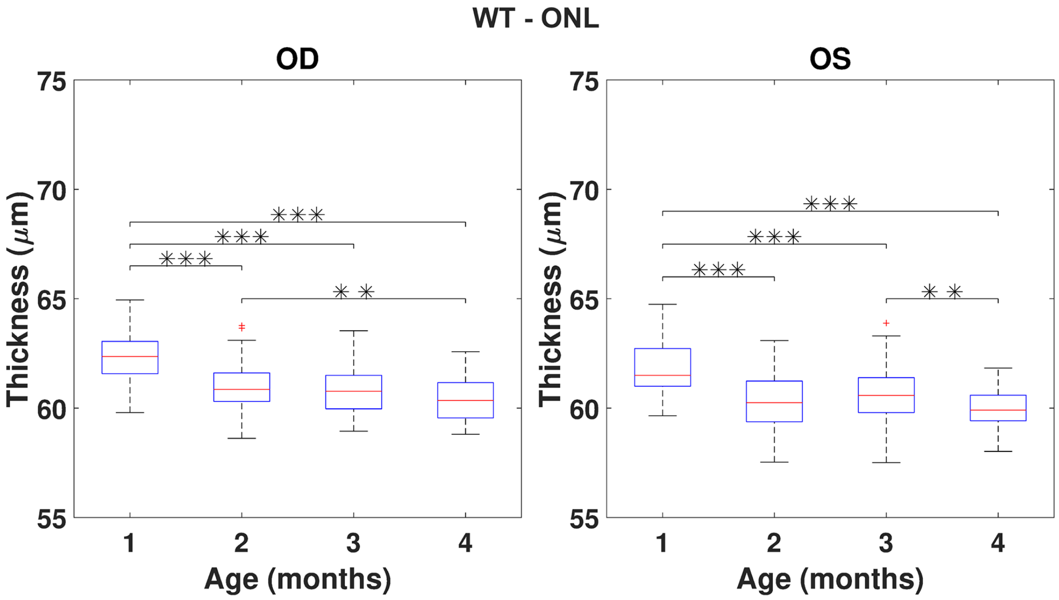

Supplementary Figure 5. Boxplot of the ONL thickness (μm) for each time point, for right and left eyes of WT mice (respectively, left and right graphs). One, two and three asterisks represent, respectively, statistically significant differences at the level of 5%, 1% and 0.1%, based on pairwise comparisons.

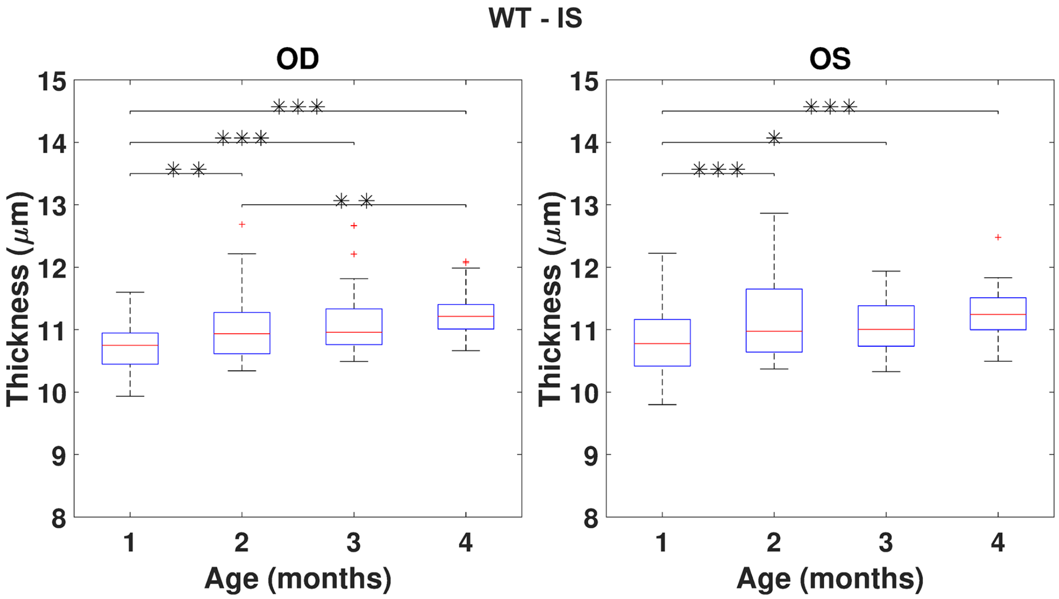

Supplementary Figure 6. Boxplot of the IS thickness (μm) for each time point, for right and left eyes of WT mice (respectively, left and right graphs). One, two and three asterisks represent, respectively, statistically significant differences at the level of 5%, 1% and 0.1%, based on pairwise comparisons.

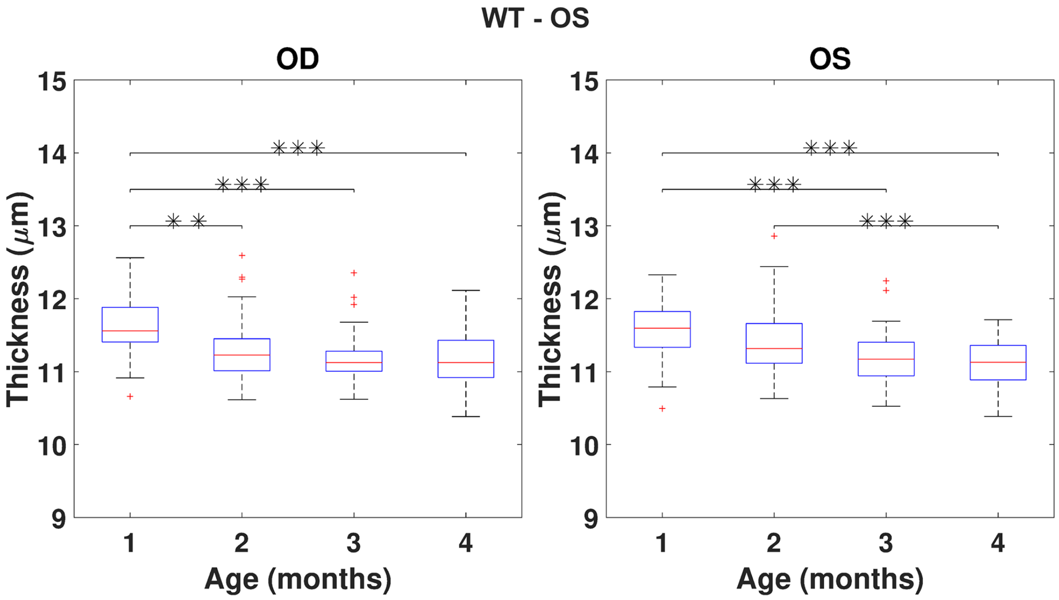

Supplementary Figure 7. Boxplot of the OS thickness (μm) for each time point, for right and left eyes of WT mice (respectively, left and right graphs). One, two and three asterisks represent, respectively, statistically significant differences at the level of 5%, 1% and 0.1%, based on pairwise comparisons.

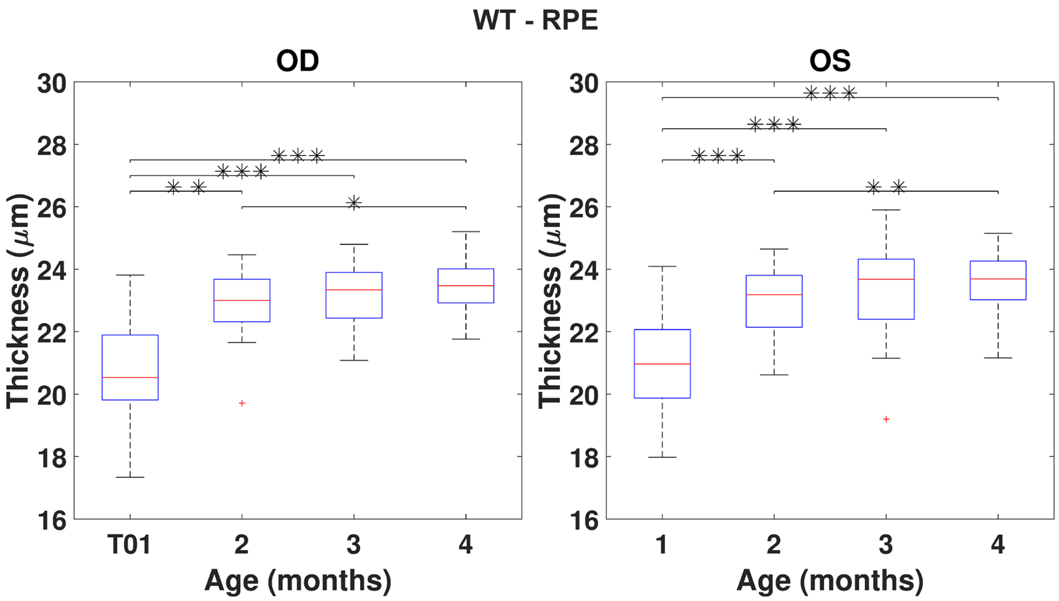

Supplementary Figure 8. Boxplot of the RPE thickness (μm) for each time point, for right and left eyes of WT mice (respectively, left and right graphs). One, two and three asterisks represent, respectively, statistically significant differences at the level of 5%, 1% and 0.1%, based on pairwise comparisons.

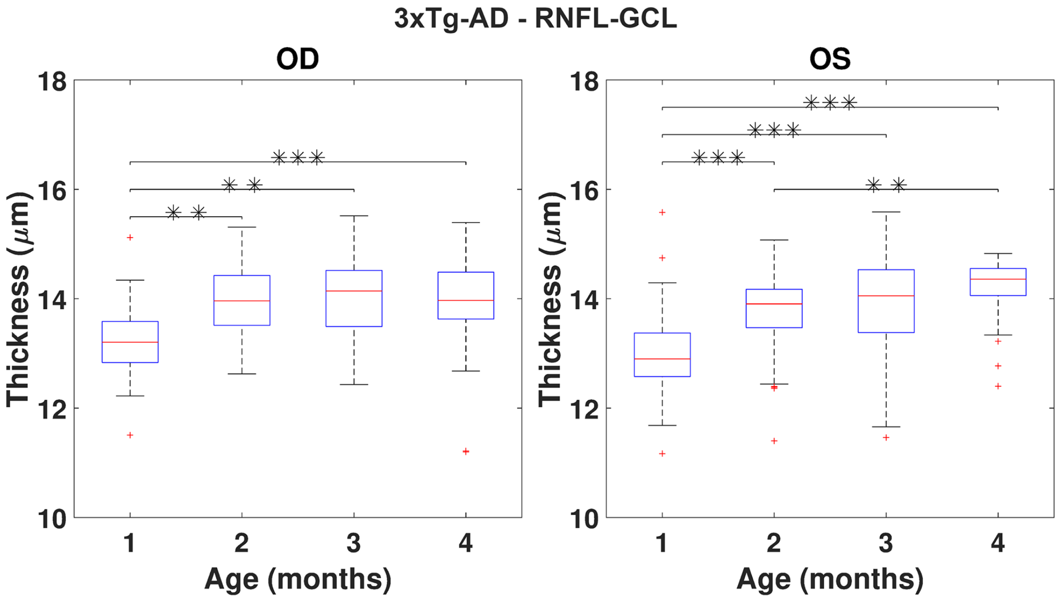

Supplementary Figure 9. Boxplot of the RNFL-GCL complex thickness (μm) for each time point, for right and left eyes of 3×Tg-AD mice (respectively, left and right graphs). One, two and three asterisks represent, respectively, statistically significant differences at the level of 5%, 1% and 0.1%, based on pairwise comparisons.

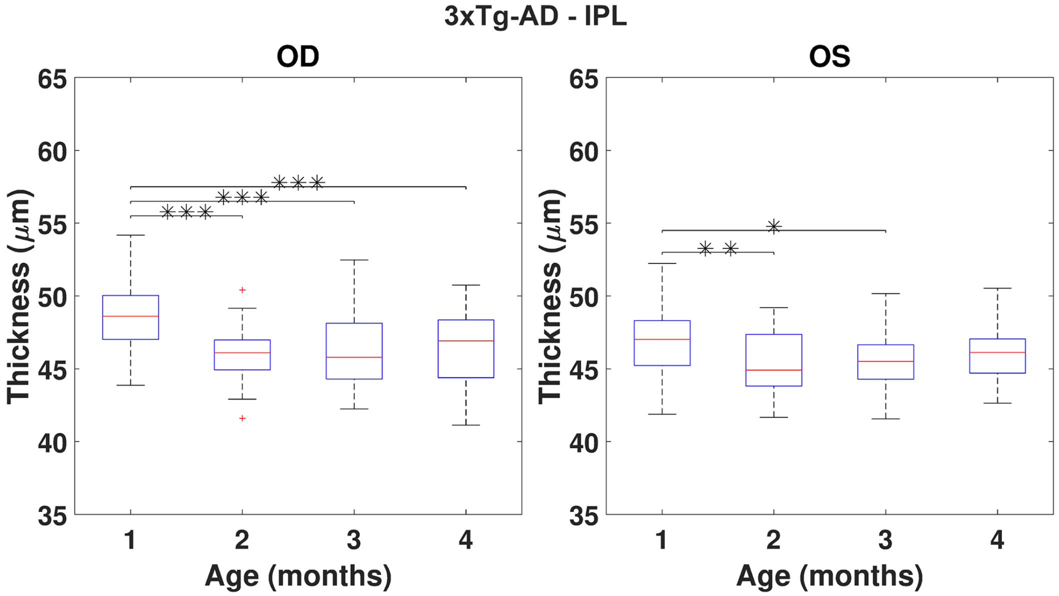

Supplementary Figure 10. Boxplot of the IPL thickness (μm) for each time point, for right and left eyes of 3×Tg-AD mice (respectively, left and right graphs). One, two and three asterisks represent, respectively, statistically significant differences at the level of 5%, 1% and 0.1%, based on pairwise comparisons.

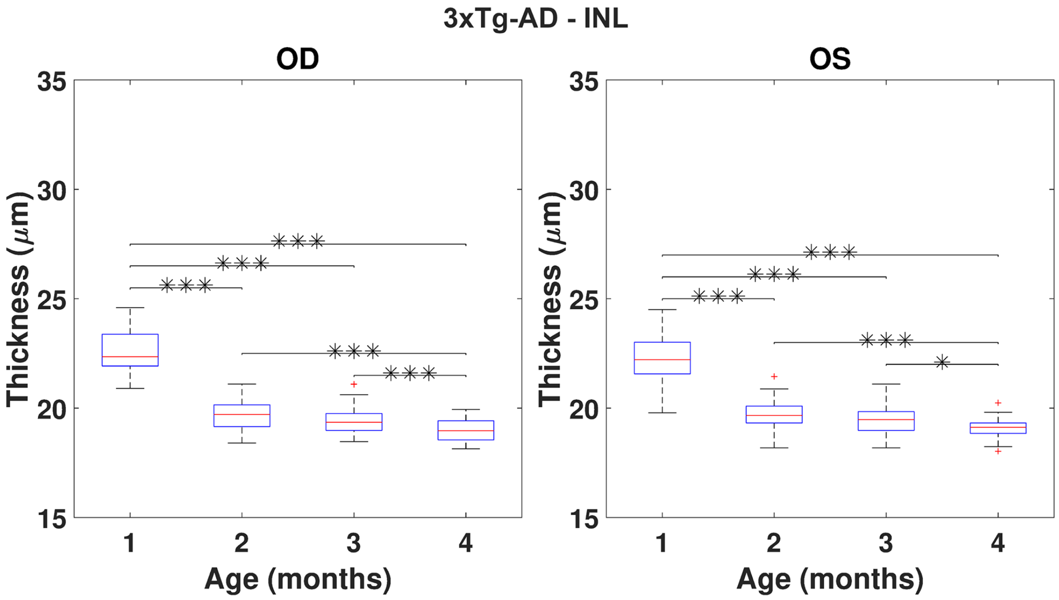

Supplementary Figure 11. Boxplot of the INL thickness (µm) for each time point, for right and left eyes of 3×Tg-AD mice (respectively, left and right graphs). One, two and three asterisks represent, respectively, statistically significant differences at the level of 5%, 1% and 0.1%, based on pairwise comparisons.

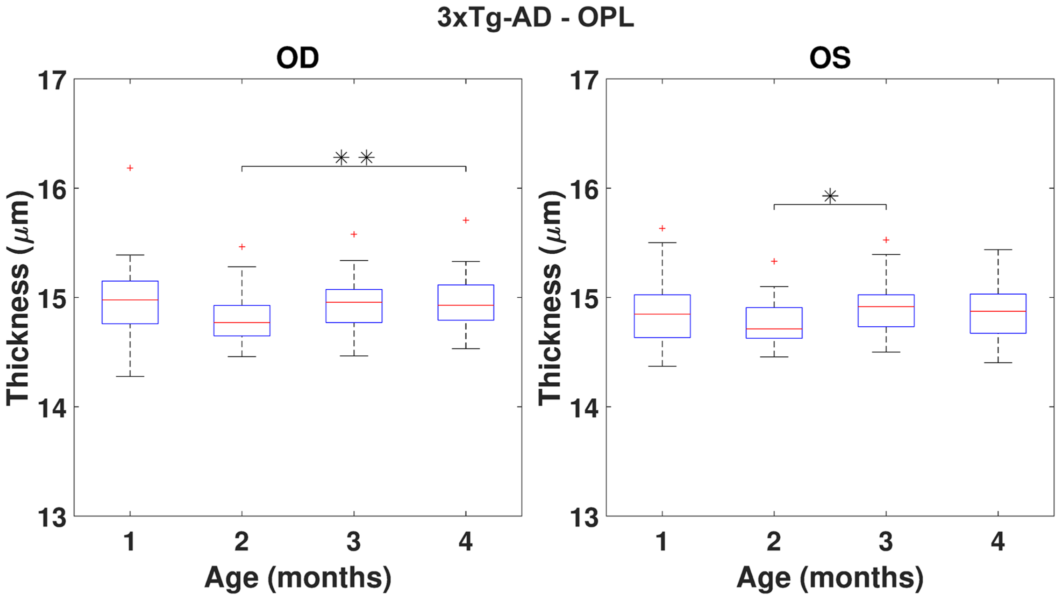

Supplementary Figure 12. Boxplot of the OPL thickness (µm) for each time point, for right and left eyes of 3×Tg-AD mice (respectively, left and right graphs). One, two and three asterisks represent, respectively, statistically significant differences at the level of 5%, 1% and 0.1%, based on pairwise comparisons.

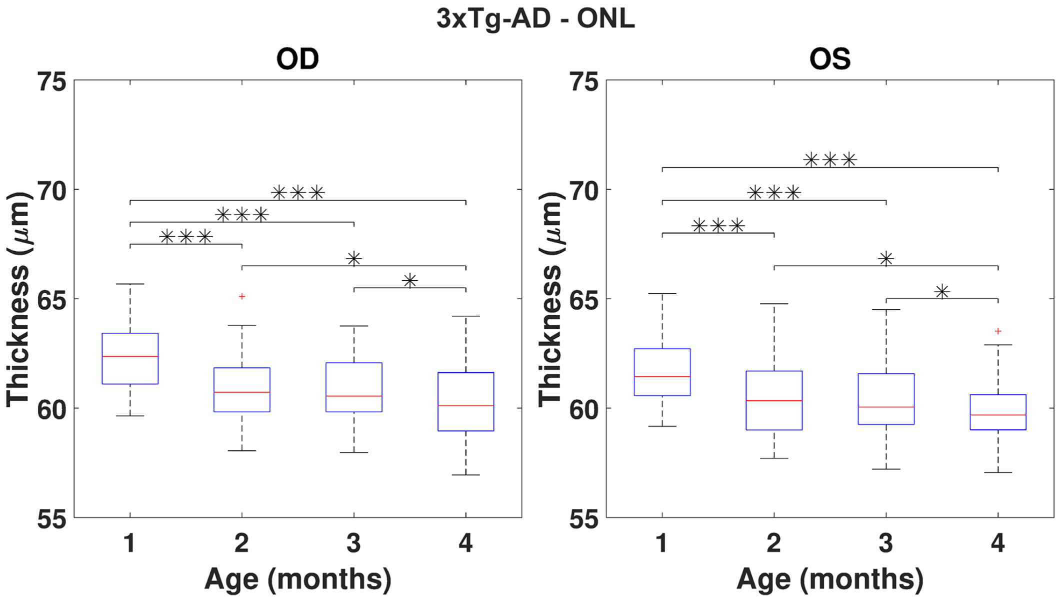

Supplementary Figure 13. Boxplot of the ONL thickness (μm) for each time point, for right and left eyes of 3×Tg-AD mice (respectively, left and right graphs). One, two and three asterisks represent, respectively, statistically significant differences at the level of 5%, 1% and 0.1%, based on pairwise comparisons.

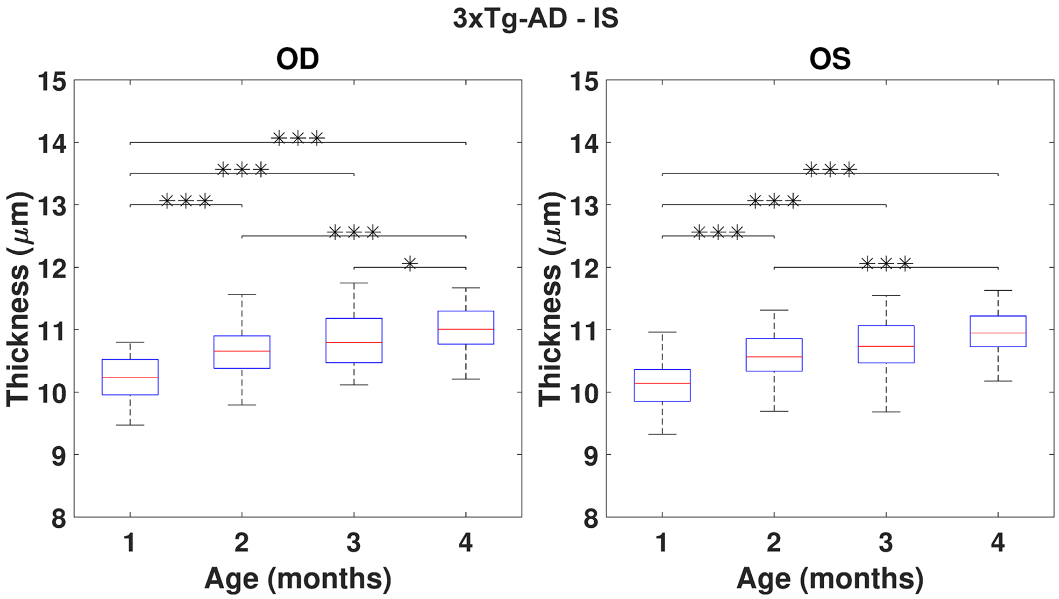

Supplementary Figure 14. Boxplot of the IS thickness (μm) for each time point, for right and left eyes of 3×Tg-AD mice (respectively, left and right graphs). One, two and three asterisks represent, respectively, statistically significant differences at the level of 5%, 1% and 0.1%, based on pairwise comparisons.

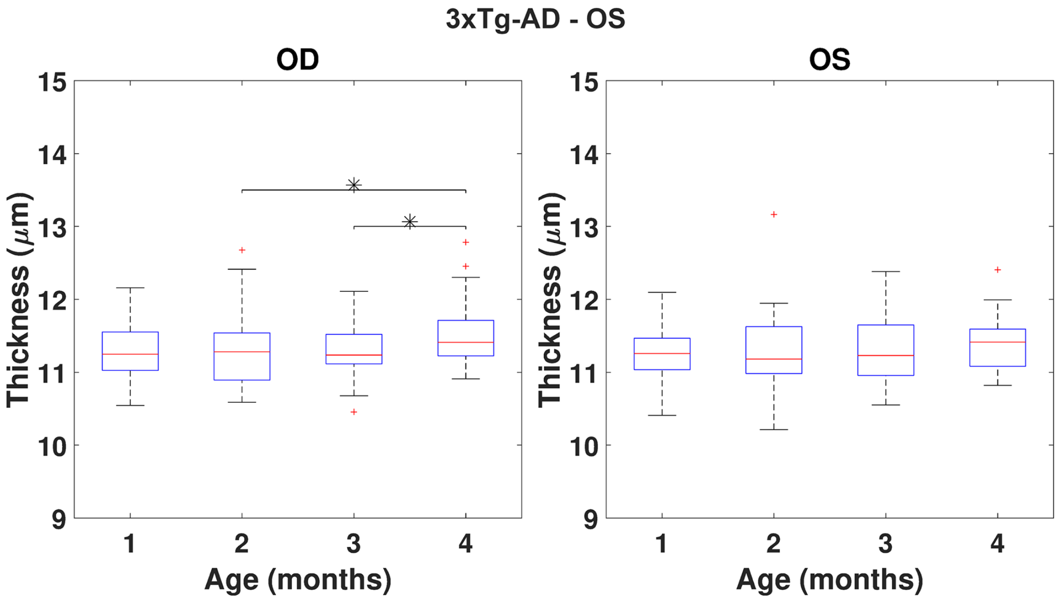

Supplementary Figure 15. Boxplot of the OS thickness (µm) for each time point, for right and left eyes of 3×Tg-AD mice (respectively, left and right graphs). One, two and three asterisks represent, respectively, statistically significant differences at the level of 5%, 1% and 0.1%, based on pairwise comparisons.

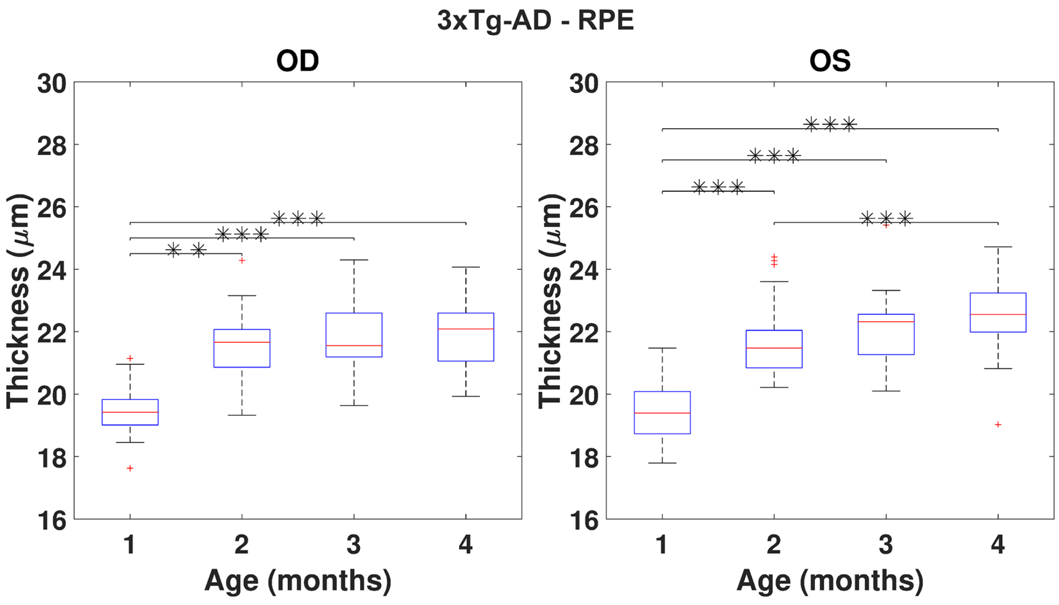

Supplementary Figure 16. Boxplot of the RPE thickness (µm) for each time point, for right and left eyes of 3×Tg-AD mice (respectively, left and right graphs). One, two and three asterisks represent, respectively, statistically significant differences at the level of 5%, 1% and 0.1%, based on pairwise comparisons.
