# Supplementary material for: Longitudinal normative OCT retinal thickness data for wild-type mice, and characterization of changes in the 3×Tg-AD mice model of Alzheimer's disease
Source: Aging (Albany NY). 2021 Apr 2;13(7):9433–54. doi: 10.18632/aging.202916 (PMC8064224; doi:10.18632/aging.202916)
Supplement: Supplementary Tables 1 to 4 [file aging-13-202916-s002.pdf]

SUPPLEMENTARY TABLES

Please browse Full Text version to see the data of Supplementary Tables 5–8.

Mean thickness

Supplementary Table 1. Thickness values (m(sd)) (in µm) for the right eyes of wild-type mice, at the ages of one, two, three and four-months-old.

	One month (N=54)	Two months (N=52)	Three months (N=49)	Four months (N=53)
RNFL-GCL	12.98 (0.67)	13.37 (0.98)	13.44 (0.93)	13.56 (0.69)
IPL	51.97 (1.99)	48.67 (2.34)	47.59 (3.18)	47.54 (1.66)
INL	25.54 (0.97)	21.90 (0.90)	21.37 (0.79)	20.56 (0.56)
OPL	15.21 (0.29)	15.15 (0.28)	15.15 (0.22)	15.14 (0.25)
ONL	62.34 (1.02)	61.00 (1.41)	60.75 (0.98)	60.38 (0.96)
IS	10.75 (0.48)	10.99 (0.51)	11.05 (0.44)	11.22 (0.41)
OS	11.63 (0.40)	11.35 (0.49)	11.19 (0.33)	11.17 (0.42)
RPE	20.87 (1.51)	22.86 (1.05)	23.14 (1.11)	23.38 (0.76)
TRT	210.39 (2.77)	203.35 (3.48)	203.03 (3.13)	200.82 (2.37)

Supplementary Table 2. Thickness values (m(sd)) (in µm) for the left eyes of wild-type mice, at the ages of one, two, three and four-months-old.

	One month (N=50)	Two months (N=50)	Three months (N=36)	Four months (N=50)
RNFL-GCL	12.81 (0.77)	12.83 (0.71)	13.44 (0.93)	13.41 (0.75)
IPL	51.11 (2.03)	47.42 (2.11)	47.46 (3.23)	46.92 (2.11)
INL	25.59 (1.08)	21.96 (0.82)	21.52 (0.99)	20.68 (0.66)
OPL	15.23 (0.32)	15.11 (0.33)	15.10 (0.20)	15.09 (0.22)
ONL	61.90 (1.28)	60.27 (1.27)	60.52 (1.38)	59.92 (0.96)
IS	10.84 (0.58)	11.15 (0.61)	11.04 (0.41)	11.19 (0.43)
OS	11.58 (0.41)	11.38 (0.45)	11.19 (0.33)	11.10 (0.36)
RPE	21.00 (1.39)	22.91 (1.03)	23.34 (1.35)	23.57 (1.02)
TRT	209.72 (3.39)	202.44 (3.05)	203.65 (4.01)	200.56 (2.45)

Supplementary Table 3. Thickness values (m(sd)) (in µm) for the right eyes of 3×Tg-AD mice, at the ages of one, two, three and four-months-old.

	One month (N=46)	Two months (N=41)	Three months (N=43)	Four months (N=40)
RNFL-GCL	13.21 (0.63)	13.89 (0.75)	14.00 (0.79)	13.91 (0.85)
IPL	48.72 (2.31)	45.92 (2.73)	46.20 (2.35)	45.95 (3.29)
INL	22.55 (0.95)	19.58 (1.01)	19.52 (0.64)	18.89 (0.96)
OPL	14.96 (0.33)	14.82 (0.30)	14.93 (0.23)	14.93 (0.31)
ONL	62.33 (1.63)	60.73 (1.94)	60.81 (1.55)	60.08 (2.12)
IS	10.22 (0.39)	10.60 (0.39)	10.82 (0.40)	11.00 (0.39)
OS	11.30 (0.41)	11.33 (0.50)	11.32 (0.39)	11.50 (0.43)
RPE	19.40 (0.74)	21.29 (1.06)	21.72 (0.97)	21.95 (0.98)
TRT	201.44 (3.77)	196.97 (4.44)	197.99 (3.28)	196.07 (3.79)

Supplementary Table 4. Thickness values (m(sd)) (in µm) for the left eyes of 3×Tg-AD mice, at the ages of one, two, three and four-months-old.

	One month (N=48)	Two months (N=42)	Three months (N=45)	Four months (N=44)
RNFL-GCL	13.10 (0.92)	13.51 (0.94)	13.90 (0.90)	14.14 (0.56)
IPL	47.14 (2.36)	45.28 (2.00)	45.43 (2.21)	45.78 (2.08)
INL	22.28 (1.02)	19.67 (0.67)	19.49 (0.61)	19.02 (0.44)
OPL	14.85 (0.28)	14.80 (0.22)	14.91 (0.23)	14.86 (0.23)
ONL	61.86 (1.52)	60.43 (1.66)	60.40 (1.70)	59.66 (1.54)
IS	10.14 (0.34)	10.62 (0.37)	10.78 (0.40)	10.94 (0.34)
OS	11.28 (0.36)	11.35 (0.51)	11.33 (0.42)	11.37 (0.37)
RPE	19.44 (0.89)	21.62 (1.04)	22.06 (1.03)	22.59 (0.97)
TRT	199.68 (3.71)	196.59 (2.86)	197.41 (3.10)	196.25 (2.45)

Supplementary Table 5. Thickness values (m(sd)) (in µm) for each block, for the right (OD) and left (OS) eyes separately, as well as thickness values obtained by combining both eyes' data (OD+OS) of WT mice at the age of one-month-old.

Supplementary Table 6. Thickness values (m(sd)) (in µm) for each block, for the right (OD) and left (OS) eyes separately, as well as thickness values obtained by combining both eyes' data (OD+OS) of WT mice at the age of two-months-old.

Supplementary Table 7. Thickness values (m(sd)) (in µm) for each block, for the right (OD) and left (OS) eyes separately, as well as thickness values obtained by combining both eyes' data (OD+OS) of WT mice at the age of three-months-old.

Supplementary Table 8. Thickness values (m(sd)) (in µm) for each block, for the right (OD) and left (OS) eyes separately, as well as thickness values obtained by combining both eyes' data (OD+OS) of WT mice at the age of four-months-old.
