# Supplementary material for: Longitudinal normative OCT retinal thickness data for wild-type mice, and characterization of changes in the 3×Tg-AD mice model of Alzheimer's disease
Source: Aging (Albany NY). 2021 Apr 2;13(7):9433–54. doi: 10.18632/aging.202916 (PMC8064224; doi:10.18632/aging.202916)
Supplement: Supplementary Table 5 [file aging-13-202916-s003.docx]

**Supplementary Table 5. Thickness values (m(sd)) (in µm) for each block, for the right (OD) and left (OS) eyes separately, as well as thickness values obtained by combining both eyes' data (OD+OS) of WT mice at the age of one-month-old.**

|  |  | Block 1 | Block 2 | Block 3 | Block 4 | Block 5 | Block 6 | Block 7 | Block 8 | Block 9 |
| --- | --- | --- | --- | --- | --- | --- | --- | --- | --- | --- |
| Right Eyes (OD) | RNFL-GCL | 11.36 (0.80) | 11.29 (0.74) | 11.41 (0.60) | 12.99 (0.82) | 13.06 (1.01) | 13.07 (0.88) | 14.41 (1.15) | 13.70 (1.28) | 14.00 (0.97) |
|  | IPL | 44.64 (2.19) | 45.36 (2.12) | 46.98 (2.28) | 52.14 (2.14) | 51.89 (2.07) | 52.16 (2.12) | 56.49 (2.01) | 56.18 (2.21) | 55.12 (2.09) |
|  | INL | 22.50 (1.15) | 23.20 (1.22) | 24.41 (1.46) | 26.05 (1.13) | 26.83 (1.15) | 27.44 (1.10) | 26.70 (0.88) | 26.31 (1.26) | 26.40 (1.27) |
|  | OPL | 15.32 (0.41) | 15.28 (0.40) | 15.42 (0.49) | 15.26 (0.29) | 15.17 (0.32) | 15.36 (0.40) | 15.14 (0.34) | 14.89 (0.29) | 15.05 (0.27) |
|  | ONL | 60.55 (1.34) | 61.41 (1.18) | 61.60 (1.37) | 62.63 (1.20) | 63.17 (1.13) | 63.15 (1.32) | 63.23 (0.99) | 62.79 (1.03) | 62.39 (1.23) |
|  | IS | 10.99 (0.59) | 10.91 (0.57) | 11.06 (0.47) | 10.80 (0.58) | 10.77 (0.57) | 10.91 (0.48) | 10.36 (0.61) | 10.32 (0.60) | 10.53 (0.50) |
|  | OS | 11.48 (0.39) | 11.51 (0.38) | 11.76 (0.38) | 11.89 (0.40) | 11.90 (0.41) | 11.92 (0.44) | 11.51 (0.46) | 11.28 (0.51) | 11.30 (0.52) |
|  | RPE | 21.48 (1.77) | 21.11 (1.67) | 21.09 (1.72) | 21.10 (1.66) | 21.19 (1.49) | 20.86 (1.53) | 20.40 (1.59) | 20.13 (1.58) | 20.25 (1.61) |
|  | TRT | 198.14 (3.26) | 199.97 (3.33) | 203.56 (4.22) | 212.89 (3.58) | 214.12 (3.62) | 214.89 (3.63) | 218.29 (3.34) | 215.74 (3.43) | 215.13 (3.29) |
| Left Eyes (OS) | RNFL-GCL | 11.54 (0.77) | 11.25 (0.72) | 11.14 (0.73) | 12.81 (0.88) | 13.02 (1.12) | 12.99 (1.02) | 14.26 (1.12) | 13.78 (1.22) | 14.08 (1.09) |
|  | IPL | 46.77 (2.17) | 45.75 (2.36) | 44.31 (1.77) | 52.23 (1.65) | 51.55 (2.12) | 51.33 (2.28) | 54.35 (2.02) | 55.03 (2.00) | 56.06 (2.39) |
|  | INL | 24.13 (1.46) | 23.15 (1.35) | 22.73 (1.27) | 27.00 (1.44) | 26.97 (1.27) | 26.53 (1.20) | 26.45 (1.22) | 26.48 (1.30) | 26.97 (1.31) |
|  | OPL | 15.42 (0.45) | 15.29 (0.43) | 15.37 (0.47) | 15.35 (0.40) | 15.17 (0.35) | 15.26 (0.39) | 15.11 (0.30) | 14.85 (0.30) | 15.06 (0.31) |
|  | ONL | 61.00 (1.76) | 60.93 (1.74) | 60.07 (1.72) | 62.69 (1.39) | 62.77 (1.36) | 62.30 (1.51) | 62.46 (1.16) | 62.39 (1.26) | 62.59 (1.43) |
|  | IS | 11.16 (0.65) | 11.03 (0.71) | 11.10 (0.65) | 10.91 (0.65) | 10.84 (0.66) | 10.94 (0.55) | 10.38 (0.70) | 10.34 (0.72) | 10.60 (0.64) |
|  | OS | 11.66 (0.40) | 11.44 (0.39) | 11.45 (0.37) | 11.83 (0.47) | 11.82 (0.46) | 11.91 (0.44) | 11.28 (0.53) | 11.21 (0.52) | 11.49 (0.50) |
|  | RPE | 21.39 (1.69) | 21.32 (1.55) | 21.93 (1.61) | 20.98 (1.43) | 21.16 (1.53) | 21.22 (1.48) | 20.33 (1.64) | 20.23 (1.62) | 20.29 (1.54) |
|  | TRT | 202.76 (4.34) | 199.67 (4.41) | 197.85 (3.79) | 213.85 (3.70) | 213.27 (3.92) | 212.50 (4.09) | 214.96 (3.97) | 214.65 (4.68) | 217.47 (4.22) |
| Combined Data (OD+OS) | RNFL-GCL | 11.45 (0.79) | 11.27 (0.72) | 11.28 (0.68) | 12.90 (0.85) | 13.04 (1.06) | 13.03 (0.95) | 14.34 (1.14) | 13.73 (1.24) | 14.04 (1.03) |
|  | IPL | 45.71 (2.42) | 45.55 (2.24) | 45.65 (2.43) | 52.18 (1.91) | 51.73 (2.09) | 51.76 (2.23) | 55.50 (2.27) | 55.64 (2.18) | 55.56 (2.27) |
|  | INL | 23.28 (1.54) | 23.18 (1.28) | 23.60 (1.61) | 26.51 (1.37) | 26.90 (1.20) | 27.00 (1.23) | 26.58 (1.06) | 26.40 (1.28) | 26.67 (1.31) |
|  | OPL | 15.37 (0.43) | 15.28 (0.41) | 15.40 (0.48) | 15.30 (0.35) | 15.17 (0.34) | 15.31 (0.40) | 15.13 (0.32) | 14.87 (0.29) | 15.06 (0.29) |
|  | ONL | 60.77 (1.56) | 61.18 (1.49) | 60.87 (1.72) | 62.66 (1.29) | 62.98 (1.25) | 62.74 (1.47) | 62.86 (1.14) | 62.60 (1.16) | 62.48 (1.32) |
|  | IS | 11.07 (0.62) | 10.97 (0.64) | 11.08 (0.56) | 10.85 (0.61) | 10.80 (0.61) | 10.93 (0.51) | 10.37 (0.65) | 10.33 (0.66) | 10.57 (0.57) |
|  | OS | 11.56 (0.40) | 11.47 (0.39) | 11.61 (0.41) | 11.86 (0.43) | 11.86 (0.44) | 11.92 (0.44) | 11.40 (0.50) | 11.25 (0.52) | 11.39 (0.52) |
|  | RPE | 21.44 (1.72) | 21.21 (1.61) | 21.50 (1.71) | 21.04 (1.55) | 21.18 (1.50) | 21.03 (1.51) | 20.37 (1.61) | 20.18 (1.59) | 20.27 (1.57) |
|  | TRT | 200.38 (4.45) | 199.82 (3.87) | 200.79 (4.92) | 213.35 (3.65) | 213.71 (3.78) | 213.73 (4.03) | 216.68 (4.01) | 215.21 (4.10) | 216.27 (3.93) |
