# Supplementary material for: Longitudinal normative OCT retinal thickness data for wild-type mice, and characterization of changes in the 3×Tg-AD mice model of Alzheimer's disease
Source: Aging (Albany NY). 2021 Apr 2;13(7):9433–54. doi: 10.18632/aging.202916 (PMC8064224; doi:10.18632/aging.202916)
Supplement: Supplementary Table 6 [file aging-13-202916-s004.docx]

**Supplementary Table 6. Thickness values (m(sd)) (in µm) for each block, for the right (OD) and left (OS) eyes separately, as well as thickness values obtained by combining both eyes' data (OD+OS) of WT mice at the age of two-months-old.**

|  |  | Block 1 | Block 2 | Block 3 | Block 4 | Block 5 | Block 6 | Block 7 | Block 8 | Block 9 |
| --- | --- | --- | --- | --- | --- | --- | --- | --- | --- | --- |
| Right Eyes (OD) | RNFL-GCL | 12.54 (1.17) | 12.13 (1.02) | 11.75 (0.68) | 13.28 (0.99) | 12.77 (1.12) | 12.73 (1.06) | 13.97 (0.97) | 13.87 (0.74) | 14.04 (0.70) |
|  | IPL | 40.24 (1.68) | 41.59 (2.00) | 42.26 (1.26) | 47.26 (2.21) | 48.12 (2.08) | 48.24 (2.07) | 51.84 (1.80) | 51.53 (1.97) | 50.71 (2.31) |
|  | INL | 18.79 (0.83) | 19.23 (1.03) | 20.58 (1.07) | 22.07 (1.03) | 22.63 (1.08) | 23.40 (1.12) | 23.63 (0.94) | 23.37 (0.95) | 23.37 (0.92) |
|  | OPL | 15.17 (0.58) | 15.16 (0.58) | 15.41 (0.51) | 15.08 (0.29) | 15.07 (0.32) | 15.38 (0.40) | 15.04 (0.25) | 14.88 (0.22) | 15.08 (0.25) |
|  | ONL | 57.38 (1.34) | 58.56 (1.46) | 59.17 (1.63) | 61.19 (1.30) | 62.08 (1.48) | 62.10 (1.44) | 62.71 (1.28) | 62.77 (1.30) | 62.17 (1.38) |
|  | IS | 11.23 (0.84) | 11.20 (0.87) | 11.48 (0.77) | 10.77 (0.58) | 10.82 (0.55) | 11.23 (0.51) | 10.62 (0.59) | 10.56 (0.49) | 10.99 (0.45) |
|  | OS | 11.22 (0.46) | 11.28 (0.49) | 11.57 (0.45) | 11.42 (0.42) | 11.36 (0.42) | 11.61 (0.48) | 11.30 (0.73) | 11.13 (0.95) | 11.27 (1.02) |
|  | RPE | 24.30 (1.69) | 22.81 (1.22) | 23.44 (1.46) | 22.92 (1.10) | 22.97 (1.26) | 22.92 (1.06) | 22.30 (1.31) | 21.90 (1.47) | 22.09 (1.46) |
|  | TRT | 190.76 (3.16) | 191.99 (3.22) | 196.17 (3.90) | 204.20 (3.94) | 205.97 (3.87) | 207.75 (3.54) | 211.76 (3.89) | 210.26 (4.03) | 209.87 (4.00) |
| Left Eyes (OS) | RNFL-GCL | 11.95 (0.71) | 12.13 (1.10) | 12.01 (1.06) | 12.46 (0.53) | 12.58 (0.71) | 12.87 (0.90) | 13.86 (0.87) | 13.60 (0.99) | 13.75 (0.86) |
|  | IPL | 43.45 (2.54) | 42.34 (1.90) | 40.53 (1.84) | 48.25 (2.04) | 47.56 (1.93) | 46.80 (2.23) | 50.22 (2.07) | 50.66 (2.22) | 51.95 (2.63) |
|  | INL | 20.40 (1.19) | 19.27 (1.12) | 18.98 (0.98) | 23.24 (1.11) | 22.77 (1.13) | 22.30 (1.01) | 23.31 (0.79) | 23.59 (0.87) | 23.61 (0.82) |
|  | OPL | 15.31 (0.58) | 15.14 (0.67) | 15.20 (0.63) | 15.29 (0.43) | 15.06 (0.35) | 15.08 (0.30) | 15.03 (0.21) | 14.86 (0.27) | 15.00 (0.24) |
|  | ONL | 58.49 (1.79) | 57.95 (1.75) | 56.87 (1.79) | 61.50 (1.28) | 61.53 (1.40) | 60.44 (1.37) | 61.73 (1.23) | 62.09 (1.30) | 61.68 (1.27) |
|  | IS | 11.64 (0.97) | 11.48 (0.96) | 11.53 (0.84) | 11.19 (0.69) | 10.93 (0.67) | 11.10 (0.55) | 10.86 (0.50) | 10.66 (0.58) | 11.00 (0.49) |
|  | OS | 11.57 (0.57) | 11.41 (0.67) | 11.38 (0.55) | 11.62 (0.50) | 11.44 (0.50) | 11.57 (0.43) | 11.07 (0.41) | 11.01 (0.51) | 11.32 (0.54) |
|  | RPE | 24.23 (1.35) | 22.73 (1.27) | 25.33 (1.75) | 22.79 (1.06) | 22.77 (1.50) | 23.13 (1.37) | 22.07 (1.30) | 21.54 (1.59) | 21.90 (1.44) |
|  | TRT | 196.90 (4.40) | 191.89 (4.22) | 191.43 (3.43) | 206.37 (3.41) | 204.59 (3.65) | 203.45 (3.72) | 208.20 (3.15) | 208.15 (3.28) | 210.19 (3.59) |
| Combined Data (OD+OS) | RNFL-GCL | 12.17 (0.95) | 12.13 (1.06) | 11.91 (0.94) | 12.87 (0.89) | 12.67 (0.93) | 12.80 (0.98) | 13.92 (0.92) | 13.74 (0.88) | 13.90 (0.79) |
|  | IPL | 42.26 (2.74) | 42.06 (1.95) | 41.17 (1.84) | 47.77 (2.17) | 47.83 (2.02) | 47.50 (2.26) | 51.07 (2.09) | 51.12 (2.13) | 51.29 (2.53) |
|  | INL | 19.59 (1.30) | 19.25 (1.07) | 19.78 (1.30) | 22.64 (1.21) | 22.70 (1.10) | 22.86 (1.20) | 23.47 (0.88) | 23.48 (0.92) | 23.49 (0.88) |
|  | OPL | 15.24 (0.58) | 15.15 (0.62) | 15.31 (0.58) | 15.18 (0.38) | 15.07 (0.33) | 15.23 (0.38) | 15.03 (0.23) | 14.87 (0.24) | 15.04 (0.25) |
|  | ONL | 57.93 (1.67) | 58.25 (1.63) | 58.02 (2.06) | 61.34 (1.30) | 61.81 (1.46) | 61.28 (1.63) | 62.22 (1.34) | 62.43 (1.34) | 61.93 (1.34) |
|  | IS | 11.43 (0.93) | 11.34 (0.92) | 11.50 (0.80) | 10.98 (0.67) | 10.87 (0.61) | 11.17 (0.53) | 10.74 (0.55) | 10.61 (0.54) | 11.00 (0.47) |
|  | OS | 11.40 (0.54) | 11.35 (0.58) | 11.47 (0.51) | 11.52 (0.47) | 11.40 (0.46) | 11.59 (0.46) | 11.19 (0.60) | 11.07 (0.76) | 11.30 (0.81) |
|  | RPE | 24.26 (1.53) | 22.77 (1.24) | 24.39 (1.86) | 22.85 (1.08) | 22.87 (1.38) | 23.02 (1.22) | 22.19 (1.30) | 21.72 (1.53) | 21.99 (1.45) |
|  | TRT | 193.80 (4.89) | 191.94 (3.73) | 193.82 (4.37) | 205.27 (3.83) | 205.29 (3.81) | 205.62 (4.21) | 210.00 (3.95) | 209.22 (3.81) | 210.03 (3.78) |
