# Supplementary material for: Longitudinal normative OCT retinal thickness data for wild-type mice, and characterization of changes in the 3×Tg-AD mice model of Alzheimer's disease
Source: Aging (Albany NY). 2021 Apr 2;13(7):9433–54. doi: 10.18632/aging.202916 (PMC8064224; doi:10.18632/aging.202916)
Supplement: Supplementary Table 7 [file aging-13-202916-s005.docx]

**Supplementary Table 7. Thickness values (m(sd)) (in µm) for each block, for the right (OD) and left (OS) eyes separately, as well as thickness values obtained by combining both eyes' data (OD+OS) of WT mice at the age of three-months-old.**

|  |  | Block 1 | Block 2 | Block 3 | Block 4 | Block 5 | Block 6 | Block 7 | Block 8 | Block 9 |
| --- | --- | --- | --- | --- | --- | --- | --- | --- | --- | --- |
| Right Eyes (OD) | RNFL-GCL | 12.19 (1.12) | 12.13 (0.98) | 11.93 (0.70) | 13.62 (0.89) | 13.26 (0.91) | 13.03 (0.57) | 14.19 (0.91) | 14.19 (1.01) | 14.18 (0.78) |
|  | IPL | 39.26 (1.79) | 40.54 (2.34) | 41.85 (2.21) | 46.02 (1.95) | 47.20 (1.86) | 47.54 (1.88) | 50.74 (2.05) | 51.57 (2.11) | 50.21 (1.84) |
|  | INL | 18.04 (0.77) | 18.69 (0.88) | 19.73 (1.28) | 21.22 (0.81) | 21.86 (0.85) | 22.65 (0.96) | 22.86 (0.72) | 22.49 (0.79) | 22.62 (0.80) |
|  | OPL | 15.08 (0.46) | 15.08 (0.44) | 15.36 (0.49) | 15.13 (0.26) | 15.09 (0.27) | 15.40 (0.38) | 15.19 (0.25) | 14.95 (0.23) | 15.15 (0.23) |
|  | ONL | 57.19 (1.43) | 58.47 (1.46) | 59.04 (1.63) | 60.65 (1.09) | 61.76 (1.13) | 61.77 (1.27) | 62.19 (1.16) | 62.32 (1.17) | 61.67 (1.09) |
|  | IS | 11.33 (0.73) | 11.26 (0.68) | 11.48 (0.63) | 10.88 (0.56) | 10.89 (0.53) | 11.28 (0.45) | 10.83 (0.46) | 10.74 (0.43) | 11.15 (0.37) |
|  | OS | 11.11 (0.44) | 11.23 (0.49) | 11.48 (0.52) | 11.22 (0.32) | 11.20 (0.38) | 11.45 (0.35) | 11.12 (0.35) | 10.95 (0.38) | 11.07 (0.39) |
|  | RPE | 25.15 (1.72) | 23.20 (1.13) | 24.13 (1.21) | 23.40 (1.21) | 23.22 (1.18) | 23.28 (1.23) | 22.60 (1.26) | 22.17 (1.32) | 22.37 (1.29) |
|  | TRT | 189.89 (2.86) | 190.76 (3.86) | 195.21 (4.67) | 202.65 (2.62) | 204.71 (3.08) | 206.32 (3.12) | 210.03 (3.15) | 209.55 (3.21) | 208.37 (2.95) |
| Left Eyes (OS) | RNFL-GCL | 12.37 (0.93) | 12.36 (1.13) | 12.09 (1.07) | 13.24 (1.15) | 13.19 (1.09) | 13.27 (1.05) | 14.55 (1.15) | 14.16 (1.06) | 14.46 (1.00) |
|  | IPL | 43.29 (1.59) | 41.52 (1.97) | 40.14 (1.82) | 47.93 (1.66) | 46.96 (1.31) | 46.79 (1.66) | 50.21 (1.94) | 50.40 (2.28) | 51.30 (2.10) |
|  | INL | 20.58 (1.11) | 19.29 (0.99) | 18.45 (0.65) | 22.55 (1.00) | 22.33 (1.07) | 21.67 (0.83) | 22.32 (0.94) | 22.49 (0.88) | 22.61 (0.88) |
|  | OPL | 15.28 (0.37) | 15.04 (0.43) | 15.07 (0.44) | 15.31 (0.41) | 15.09 (0.26) | 15.14 (0.30) | 15.12 (0.25) | 14.87 (0.20) | 15.06 (0.26) |
|  | ONL | 59.88 (1.72) | 59.00 (1.78) | 57.36 (1.91) | 61.52 (1.38) | 61.71 (1.51) | 60.36 (1.40) | 61.12 (1.15) | 61.74 (1.46) | 61.26 (1.40) |
|  | IS | 11.34 (0.59) | 11.18 (0.73) | 11.32 (0.70) | 11.20 (0.53) | 10.93 (0.52) | 11.14 (0.44) | 10.91 (0.41) | 10.65 (0.41) | 10.96 (0.36) |
|  | OS | 11.49 (0.39) | 11.24 (0.43) | 11.26 (0.39) | 11.48 (0.42) | 11.19 (0.35) | 11.43 (0.36) | 10.99 (0.38) | 10.77 (0.38) | 11.10 (0.41) |
|  | RPE | 25.23 (1.61) | 23.75 (1.48) | 25.75 (2.16) | 23.69 (1.45) | 23.44 (1.38) | 23.52 (1.27) | 22.52 (1.47) | 22.12 (1.56) | 21.95 (1.39) |
|  | TRT | 199.40 (4.51) | 193.30 (3.99) | 191.36 (3.11) | 207.50 (3.67) | 205.58 (3.63) | 203.91 (3.22) | 208.38 (3.80) | 207.86 (4.18) | 208.92 (3.91) |
| Combined Data (OD+OS) | RNFL-GCL | 12.28 (1.02) | 12.25 (1.05) | 12.01 (0.90) | 13.44 (1.03) | 13.22 (0.99) | 13.14 (0.83) | 14.36 (1.04) | 14.17 (1.03) | 14.31 (0.89) |
|  | IPL | 41.24 (2.64) | 41.02 (2.20) | 41.01 (2.19) | 46.92 (2.05) | 47.09 (1.62) | 47.18 (1.80) | 50.49 (2.01) | 51.02 (2.26) | 50.73 (2.03) |
|  | INL | 19.30 (1.59) | 18.99 (0.98) | 19.10 (1.20) | 21.86 (1.12) | 22.09 (0.99) | 22.18 (1.02) | 22.60 (0.87) | 22.49 (0.83) | 22.61 (0.83) |
|  | OPL | 15.18 (0.43) | 15.06 (0.43) | 15.22 (0.49) | 15.22 (0.35) | 15.09 (0.27) | 15.27 (0.37) | 15.16 (0.25) | 14.91 (0.22) | 15.11 (0.24) |
|  | ONL | 58.52 (2.07) | 58.73 (1.64) | 58.21 (1.96) | 61.07 (1.31) | 61.74 (1.32) | 61.09 (1.50) | 61.68 (1.27) | 62.05 (1.34) | 61.48 (1.26) |
|  | IS | 11.33 (0.66) | 11.22 (0.71) | 11.40 (0.67) | 11.03 (0.57) | 10.91 (0.52) | 11.21 (0.45) | 10.87 (0.44) | 10.69 (0.42) | 11.06 (0.38) |
|  | OS | 11.30 (0.45) | 11.23 (0.46) | 11.37 (0.47) | 11.34 (0.39) | 11.19 (0.36) | 11.44 (0.35) | 11.06 (0.37) | 10.86 (0.39) | 11.09 (0.40) |
|  | RPE | 25.19 (1.66) | 23.47 (1.34) | 24.93 (1.92) | 23.54 (1.33) | 23.33 (1.28) | 23.40 (1.25) | 22.56 (1.36) | 22.15 (1.43) | 22.17 (1.35) |
|  | TRT | 194.58 (6.07) | 192.01 (4.11) | 193.31 (4.40) | 205.00 (3.99) | 205.14 (3.37) | 205.15 (3.38) | 209.23 (3.56) | 208.73 (3.79) | 208.63 (3.44) |
