# Supplementary material for: Longitudinal normative OCT retinal thickness data for wild-type mice, and characterization of changes in the 3×Tg-AD mice model of Alzheimer's disease
Source: Aging (Albany NY). 2021 Apr 2;13(7):9433–54. doi: 10.18632/aging.202916 (PMC8064224; doi:10.18632/aging.202916)
Supplement: Supplementary Table 8 [file aging-13-202916-s006.docx]

**Supplementary Table 8: Thickness values (m(sd)) (in µm) for each block, for the right (OD) and left (OS) eyes separately, as well as thickness values obtained by combining both eyes' data (OD+OS) of WT mice at the age of four-months-old.**

|  |  | Block 1 | Block 2 | Block 3 | Block 4 | Block 5 | Block 6 | Block 7 | Block 8 | Block 9 |
| --- | --- | --- | --- | --- | --- | --- | --- | --- | --- | --- |
| Right Eyes (OD) | RNFL-GCL | 12.50 (1.28) | 12.29 (1.11) | 11.96 (0.72) | 13.76 (0.92) | 12.98 (0.93) | 12.81 (0.69) | 14.40 (0.80) | 14.46 (0.84) | 14.29 (0.66) |
|  | IPL | 40.18 (1.87) | 40.85 (1.79) | 42.55 (2.10) | 46.63 (2.01) | 46.60 (1.45) | 47.24 (1.46) | 50.58 (1.55) | 50.41 (1.83) | 49.49 (1.76) |
|  | INL | 17.60 (0.51) | 18.27 (0.79) | 19.59 (0.86) | 20.56 (0.66) | 21.39 (0.80) | 22.21 (0.79) | 22.10 (0.77) | 21.73 (0.92) | 21.63 (0.73) |
|  | OPL | 14.99 (0.28) | 15.01 (0.33) | 15.29 (0.33) | 15.15 (0.32) | 15.11 (0.32) | 15.42 (0.38) | 15.17 (0.28) | 14.99 (0.28) | 15.14 (0.23) |
|  | ONL | 57.14 (1.18) | 58.36 (1.13) | 59.16 (1.23) | 60.45 (1.13) | 61.73 (1.06) | 61.60 (1.18) | 61.99 (1.20) | 62.00 (1.20) | 61.01 (1.26) |
|  | IS | 11.44 (0.53) | 11.41 (0.55) | 11.69 (0.53) | 11.00 (0.50) | 11.07 (0.53) | 11.55 (0.46) | 10.80 (0.42) | 10.73 (0.40) | 11.29 (0.39) |
|  | OS | 11.17 (0.52) | 11.31 (0.58) | 11.57 (0.63) | 11.18 (0.40) | 11.11 (0.43) | 11.40 (0.49) | 10.97 (0.36) | 10.82 (0.38) | 11.05 (0.37) |
|  | RPE | 25.62 (2.09) | 22.97 (1.06) | 24.56 (1.34) | 23.34 (1.03) | 23.23 (1.34) | 23.32 (1.02) | 22.79 (1.15) | 22.17 (1.30) | 22.38 (1.20) |
|  | TRT | 189.47 (2.74) | 189.55 (3.22) | 195.61 (4.06) | 201.77 (2.90) | 203.17 (2.69) | 205.47 (2.97) | 208.81 (3.11) | 207.29 (3.73) | 206.24 (3.41) |
| Left Eyes (OS) | RNFL-GCL | 12.13 (0.92) | 12.13 (1.32) | 11.80 (1.35) | 12.87 (0.69) | 12.92 (0.80) | 13.32 (0.86) | 14.46 (0.80) | 14.17 (0.70) | 14.47 (0.68) |
|  | IPL | 42.85 (1.37) | 41.80 (1.52) | 39.86 (1.65) | 47.28 (1.53) | 47.03 (1.18) | 46.23 (1.67) | 49.03 (1.50) | 49.46 (1.40) | 50.48 (1.87) |
|  | INL | 19.72 (0.93) | 18.44 (0.78) | 17.94 (0.53) | 22.18 (0.88) | 21.62 (0.78) | 21.02 (0.77) | 21.59 (0.75) | 21.62 (0.78) | 21.85 (0.87) |
|  | OPL | 15.23 (0.34) | 14.96 (0.33) | 15.03 (0.31) | 15.34 (0.37) | 15.06 (0.33) | 15.11 (0.26) | 15.08 (0.23) | 14.89 (0.23) | 15.08 (0.25) |
|  | ONL | 59.15 (1.44) | 58.12 (1.34) | 56.84 (1.57) | 61.34 (1.12) | 61.33 (1.15) | 60.07 (1.32) | 60.46 (0.95) | 61.19 (1.02) | 60.62 (0.96) |
|  | IS | 11.61 (0.69) | 11.43 (0.63) | 11.51 (0.57) | 11.33 (0.58) | 11.01 (0.55) | 11.17 (0.45) | 11.01 (0.37) | 10.67 (0.40) | 11.05 (0.33) |
|  | OS | 11.45 (0.52) | 11.29 (0.49) | 11.25 (0.44) | 11.20 (0.42) | 11.02 (0.42) | 11.24 (0.38) | 10.89 (0.38) | 10.65 (0.36) | 10.93 (0.37) |
|  | RPE | 25.67 (1.40) | 23.44 (1.35) | 27.35 (2.30) | 23.67 (1.23) | 23.04 (1.57) | 23.71 (1.19) | 22.12 (1.58) | 21.41 (1.92) | 21.85 (1.50) |
|  | TRT | 197.47 (3.92) | 190.95 (3.45) | 191.12 (2.87) | 205.17 (3.05) | 202.98 (2.93) | 201.93 (2.92) | 204.59 (2.73) | 204.04 (2.85) | 206.42 (3.18) |
| Combined Data (OD+OS) | RNFL-GCL | 12.29 (1.10) | 12.20 (1.22) | 11.87 (1.11) | 13.31 (0.92) | 12.95 (0.86) | 13.06 (0.82) | 14.43 (0.80) | 14.32 (0.79) | 14.38 (0.67) |
|  | IPL | 41.67 (2.08) | 41.38 (1.69) | 41.05 (2.28) | 46.96 (1.81) | 46.82 (1.33) | 46.73 (1.64) | 49.82 (1.71) | 49.95 (1.70) | 49.98 (1.87) |
|  | INL | 18.65 (1.30) | 18.36 (0.79) | 18.77 (1.10) | 21.38 (1.13) | 21.50 (0.79) | 21.61 (0.98) | 21.85 (0.80) | 21.67 (0.85) | 21.74 (0.81) |
|  | OPL | 15.11 (0.34) | 14.99 (0.33) | 15.16 (0.35) | 15.25 (0.36) | 15.08 (0.32) | 15.26 (0.36) | 15.13 (0.26) | 14.94 (0.26) | 15.11 (0.24) |
|  | ONL | 58.13 (1.65) | 58.24 (1.24) | 58.01 (1.82) | 60.90 (1.20) | 61.53 (1.12) | 60.83 (1.47) | 61.23 (1.32) | 61.60 (1.18) | 60.82 (1.13) |
|  | IS | 11.53 (0.61) | 11.42 (0.59) | 11.60 (0.55) | 11.17 (0.56) | 11.04 (0.54) | 11.36 (0.49) | 10.90 (0.41) | 10.70 (0.40) | 11.17 (0.38) |
|  | OS | 11.31 (0.53) | 11.30 (0.54) | 11.42 (0.56) | 11.19 (0.41) | 11.06 (0.43) | 11.32 (0.44) | 10.93 (0.37) | 10.73 (0.38) | 10.99 (0.37) |
|  | RPE | 25.64 (1.77) | 23.20 (1.23) | 25.94 (2.34) | 23.51 (1.14) | 23.14 (1.46) | 23.52 (1.12) | 22.46 (1.41) | 21.79 (1.67) | 22.12 (1.38) |
|  | TRT | 193.43 (5.24) | 190.24 (3.39) | 193.39 (4.17) | 203.47 (3.42) | 203.08 (2.80) | 203.70 (3.43) | 206.72 (3.61) | 205.68 (3.69) | - 1. 3.28) |
